# In Situ Electropolymerized Ambipolar Copolymers for Vertical OECTs

**DOI:** 10.1002/smll.202411219

**Published:** 2025-04-30

**Authors:** Roman Gańczarczyk, Magdalena Rudowska, Maciej Gryszel, Adam Proń, Renata Rybakiewicz‐Sekita, Eric D. Głowacki

**Affiliations:** ^1^ Warsaw University of Technology Faculty of Chemistry Noakowskiego 3 Warsaw 00‐664 Poland; ^2^ Bioelectronics Materials and Devices Laboratory Central European Institute of Technology Brno University of Technology Purkyňova 123 Brno 61200 Czech Republic; ^3^ Linköping University Laboratory of Organic Electronics ITN, Bredgatan 33 Norrköping 60174 Sweden; ^4^ Cardinal Stefan Wyszynski University Faculty of Mathematics and Natural Sciences School of Exact Sciences Woycickiego 1/3 Warsaw 01‐938 Poland

**Keywords:** ambipolar materials, ambipolar vertical OECT, donor–acceptor systems, electropolymerization, glycolated dithienopyrrole, in situ electrochemical deposition, molecular electronics, naphthalene diimide

## Abstract

A novel approach is reported for obtaining ambipolar electroactive polymers via in situ electropolymerization for vertical organic electrochemical transistor (*v*OECT) applications. It is shown that electropolymerization is a practical and efficient method to obtain copolymers without contamination from chemical polymerization processes. To this end, two monomers, **G‐DTP‐Bu‐NDI** and **G‐DTP‐G‐NDI**, are proposed, comprising naphthalene diimide (NDI) as the acceptor core and dithienopyrrole (DTP) as the donor unit, capable of forming carbon–carbon bonds under the influence of an electric current. The incorporation of oligo(oxyethylene) (OEG) side groups ensures their amphiphilicity. Both compounds underwent successful electrochemical polymerization, resulting in thin, porous, uniform polymer layers on the electrode surface. The synthesized polymers are further examined using electrochemical and spectroelectrochemical techniques in both organic and aqueous electrolytes. Regardless of the electrolyte medium (aqueous or non‐aqueous), poly(G‐DTP‐Bu‐NDI), and poly(G‐DTP‐G‐NDI) exhibit stable electroactivity, as demonstrated by numerous scans showing ambipolar redox behavior. Both polymers are tested as components of vertical OECTs, following in situ electrochemical deposition within a 350 nm channel. The recorded transfer characteristics suggest that the fabricated donor–acceptor (D‐A) compounds hold promise for developing a new generation of ambipolar ECT devices.

## Graphical abstract

Electropolymerization is the old‐school route to conducting polymers. For emerging applications like electrochemical transistors, using electropolymerization provides intriguing advantages, such as being able to synthesize donor–acceptor copolymers that will be difficult or impossible to process otherwise.

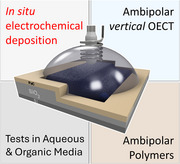

## 1 Introduction

The field of organic bioelectronics continues to grow. Many biosensing or neural interface technologies applications rely on polymers exhibiting mixed ionic‐electronic conductivity (MIEC). This property allows their use as transducer materials, efficiently interconverting electronic and ionic signals.^[^ ^1^, ^2^, ^3^ ^]^ In this perspective, ambipolar polymeric semiconductors stand out as a highly desirable group of electroactive materials. Ambipolarity can result in versatile biosensor applications, or organic electrochemical transistors (OECTs), for sensitive amplification of neuronal signals. Currently, the list of ambipolar polymers for such applications is very limited, and there is a need to find new types of polymers that combine good ambipolar conduction, stability, and ease of processability.^[^ ^4^, ^5^, ^6^, ^7^, ^8^, ^9^, ^10^, ^11^ ^]^ The primary technique to achieve ambipolarity is introducing structural elements into polymers that support electron‐accepting and electron‐donating redox reactions. This implies the utilization of donor–acceptor (D‐A) copolymers.^[^ ^5^, ^6^ ^]^ Modifying donor–acceptor interactions within this group of compounds through relatively simple structural modifications enables precise control of pivotal parameters crucial for their application.^[^ ^6^ ^]^

The application of an appropriate strategy in monomer design holds significant relevance in our study, as it facilitates the electrochemical polymerization of donor–acceptor compounds. Notably, most research on electrochemical processes predominantly focuses on donor‐type compounds, forming polymers with primarily p‐type conductivity. These polymers are commonly derivatives of polythiophenes,^[^ ^12^, ^13^, ^14^ ^]^ polypyrroles,^[^ ^15^, ^16^ ^]^ and polycarbazoles,^[^ ^17^ ^]^ widely employed in organic electronic device engineering.^[^ ^18^, ^19^, ^20^, ^21^, ^22^, ^23^ ^]^ The proposed approach enables synthesis via electropolymerization of polymers exhibiting holes and electron mobility along the main polymer chain. Electropolymerization is a well‐established method for producing thin polymer films with diverse electronic and optical properties.^[^ ^24^, ^25^, ^26^, ^27^, ^28^ ^]^ However, in the context of in situ deposition of electroactive materials for organic electronic devices, this technique remains largely underappreciated. The key advantages of electropolymerization should be emphasized: First, it offers precise control over the deposition conditions of the active material, such as monomer concentration, voltage, and current parameters, and the choice of supporting electrolyte salts, enabling the straightforward fabrication of polymer films with tailored electronic and morphological properties for specific applications.^[^ ^18^, ^19^, ^29^ ^]^ Second, this method circumvents several steps typically required in chemical polymerization, such as polymer purification, fractionation, and processing into solution‐compatible forms. Electropolymerization also permits the in situ fabrication of polymers without using metals (e.g., palladium from polymerization catalysts, see the impact of polymer purity on the performance of OECT.^[^ ^30^ ^]^) at the target location, eliminating undesirable losses associated with classical deposition methods like spin‐coating. So far, the possibility of applying electropolymerization in the fabrication of OECTs has been demonstrated only for transistors operating in p‐type mode.^[^ ^18^, ^19^, ^31^, ^32^ ^]^ Therefore, in this work, we have employed electropolymerization as an in situ method for depositing the novel ambipolar electroactive materials designed explicitly for OECT applications. A commonly utilized electron‐accepting unit is naphthalene diimide (NDI).^[^ ^33^, ^34^ ^]^ Alternating copolymers of diimide and bithiophene have found application as active layers in ambipolar organic electrochemical transistors, but only when both acceptor and donor blocks were functionalized with oligo(ethylene glycol) (OEG) chains.^[^ ^35^ ^]^ The nature of the side chains could critically affect ion injection and transport in OECT devices.^[^ ^36^ ^]^ For instance, oligoglycol side chains facilitate swelling of the polymer and, thus, more efficient ion penetration crucial for electrochemical doping in water.^[^ ^37^, ^38^ ^]^ Also, the polymers containing glycol units better stabilize positive charges imposed upon doping of the polymer backbone, which is essential for the long‐term stability of the material during the operation of OECTs.^[^ ^39^ ^]^ Nonetheless, the ratio of glycol groups to alkyl groups within the molecule can prove equally crucial, as it should not fall below 50%.^[^ ^40^, ^41^ ^]^ The significant factor determining the operational parameters of OECTs is the supramolecular organization of the polymer layer. Improved supramolecular organization can be achieved through core NDI modification.^[^ ^5^, ^42^ ^]^ Other methods involve introducing an alkyl spacer between the imide nitrogen atom in NDI and the methoxy group^[^ ^41^, ^43^, ^44^ ^]^ or replacing bithiophene with condensed donor systems such as dithienopyrrole, DTP, where α‐α’ coupled fused thiophenes rings are capable of stiffening the molecular backbone structure. DTP can be readily functionalized with an OEG chain at the pyrrolic nitrogen atom.^[^ ^45^ ^]^ Its derivatives with a unsubstituted α‐position are capable of easy electropolymerization.^[^ ^24^, ^26^, ^46^ ^]^ Moreover, polymeric products containing DTP units exhibit several desirable properties, such as electrochromism over a wide range of colors^[^ ^24^, ^26^, ^46^ ^]^ and a high capacity for charge storage.^[^ ^46^ ^]^ All these DTP advantages prompted us to harness the best properties of NDI and combine them with DTP. Herein, we develop two new compounds based on NDI functionalized with glycolated DTP moieties. We test these unique materials in aqueous environments, followed by their application in organic electrochemical transistor devices with balanced ambipolarity. Our work employed electrochemical deposition as an innovative tool for in situ polymerization within the transistor channel, demonstrating its effectiveness in producing ambipolar semiconductors for electrochemical transistor applications.

## 2 Results And Discussion

### 2.1 Synthesis

**Scheme 1:**
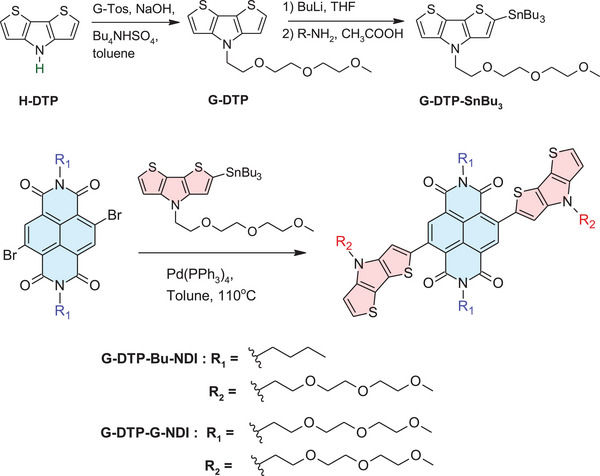
Chemical structure and synthesis of **G‐DTP‐Bu‐NDI** and **G‐DTP‐G‐NDI**.

Two new donor–acceptor–donor type compounds, namely 2,7‐dibutyl‐4,9‐bis(4‐(2‐(2‐(2‐methoxyethoxy)ethoxy)ethyl)‐4*H*‐dithieno[3,2‐*b*:2′,3′‐*d*]pyrrol‐2‐yl)benzo[*lmn*][3,8]phenanthroline‐1,3,6,8(2*H*,7*H*)‐tetraone (**G‐DTP‐Bu‐NDI**) and 2,7‐bis(2‐(2‐(2‐methoxyethoxy)ethoxy)ethyl)‐4,9‐bis(4‐(2‐(2‐(2‐methoxyethoxy)ethoxy)ethyl)‐4*H*‐dithieno[3,2‐*b*:2′,3′‐*d*]pyrrol‐2‐yl)benzo[*lmn*]^[^ ^3^, ^8^ ^]^ phenanthroline‐1,3,6,8(2*H*,7*H*)‐tetraone (**G‐DTP‐G‐NDI**) were prepared by Stille coupling, using Pd(PPh_3_)_4_ as a catalyst. In both cases, the electron donating groups were symmetrically attached to the acceptor central unit via coupling between 4‐(2‐(2‐(2‐methoxyethoxy)ethoxy)ethyl)‐2‐(tributylstannyl)‐4*H*‐dithieno[3,2‐*b*:2′,3′‐*d*]pyrrole (G‐DTP‐SnBu_3_) and an appropriate dibromo derivative of NDI. The synthetic route is presented in **Scheme** **1**. The detailed preparation procedures are described in the Supporting Information (SI), together with spectroscopic characterization of the synthesized compounds.

### 2.2 Spectroscopic, Electrochemical, and Spectroelectrochemical Characterization

**Figure 1:**
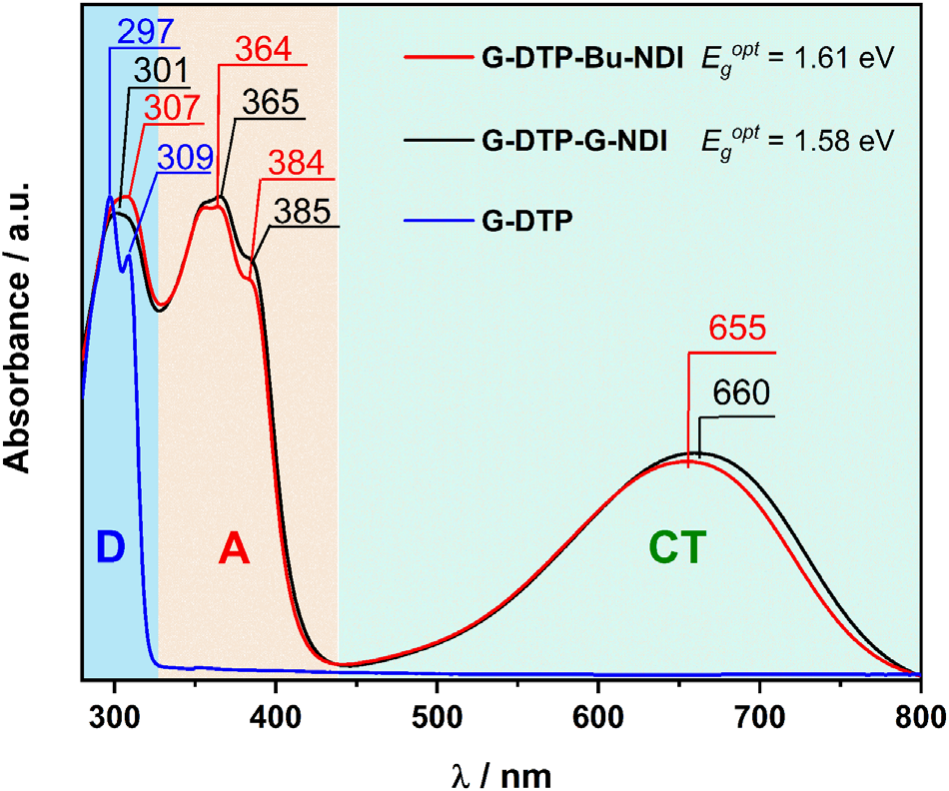
UV–vis spectra of **G‐DTP‐Bu‐NDI**, **G‐DTP‐G‐NDI** and **G‐DTP** in DCM. The values of the optical bandgap were calculated using the formula: $E_{g}^{opt}=\frac{hc}{\lambda_{onset}}\;$, where *hc*  =  1240 (*eV* · *nm*), λ_*onset*_ (*nm*) denotes the wavelength corresponding to the onset of the band on the side at the lower frequency waves. λ_*onset*_: 770 nm for **G‐DTP‐Bu‐NDI** and 783 nm for **G‐DTP‐G‐NDI**.

**Figure** **1** presents the solution spectra of the obtained diimide derivatives recorded in dichloromethane. Additionally, the spectrum of the donor unit 4‐(2‐(2‐(2‐methoxyethoxy)ethoxy)ethyl)‐4*H*‐dithieno[3,2‐*b*:2′,3′‐*d*]pyrrole (**G‐DTP**) is added to this figure to facilitate the assignment of specific bands in the spectra of the final products. From this comparative analysis, it is possible to distinguish bands originating from the donor moiety from those arising from the acceptor part of the molecule. In both cases, intense bands were observed at 307 and 301 nm for **G‐DTP‐Bu‐NDI** and **G‐DTP‐G‐NDI**, respectively, originating from the dithienopyrrole substituent. These bands closely coincide with the two bands registered in the spectrum of G‐DTP (297 and 309 nm). Furthermore, bands of distinctly vibronic character at 364 nm (*0‐1* transition) and 384 nm (*0‐0*) in the case of G‐DTP‐Bu‐NDI as well as 365 nm (*0‐1*) and 384 nm (*0‐0*) for G‐DTP‐G‐NDI, can be assigned to the π‐π* transition in the NDI core.^[^ ^26^ ^]^ In both cases, due to the conjugation between the NDI core and the electron‐donating unit, an additional broad band appears at 655 and 660 nm for **G‐DTP‐Bu‐NDI** and **G‐DTP‐G‐NDI**, respectively. This band, known as charge transfer (CT), is observed for arylene diimides substituted in the core with various donor groups.^[^ ^47^ ^]^ Its absence is noted in the case of dibromo‐naphthalene diimide with glycol chains (gNDI‐Br_2_).^[^ ^48^ ^]^ The position of the CT band reflects the strength of the donor–acceptor interactions within the molecule, undergoing a bathochromic shift with an increase in these interactions. For instance, when comparing analogous NDI derivatives with carbazole substituents,^[^ ^25^ ^]^ triarylamine,^[^ ^47^ ^]^ and dithienopyrrole substituents,^[^ ^26^ ^]^ the largest bathochromic shift of the CT band is observed in the latter case. The minor (<0.02 eV) bathochromic shift observed in the UV–vis spectrum of **G‐DTP‐G‐NDI** as compared to **G‐DTP‐Bu‐NDI** stems from the inductive effect of oxygen on the carbon atoms bonded to the NDI core. The reduced electron density on the first two carbons of the glycol chain allows expansion of the LUMO orbital area, localized mainly on NDI, effectively enhancing its electron‐accepting capacity and thereby inducing a small bathochromic shift of the CT band. A similar behavior was found for copolymeric derivatives of naphthalene‐1,4,5,8‐tetracarboxylic‐diimide and bithiophene (NDI‐T), where a branched alkyl side chain was gradually replaced by a linear ethylene glycol‐based side chain.^[^ ^41^ ^]^ With an increase in the percentage of glycol side chains (0, 10, 25, 50, 75, 90, and 100%) relative to the alkyl side chains, the CT absorption maximum shifts from 692 nm for the 0% glycol polymer to 718 nm for the 100% glycol one. Solid state spectra of **G‐DTP‐G‐NDI** and **G‐DTP‐Bu‐NDI** recorded for their thin films deposited on a quartz substrate can be found in the Supporting Information (Figure S11, Supporting Information).

**Figure 2:**
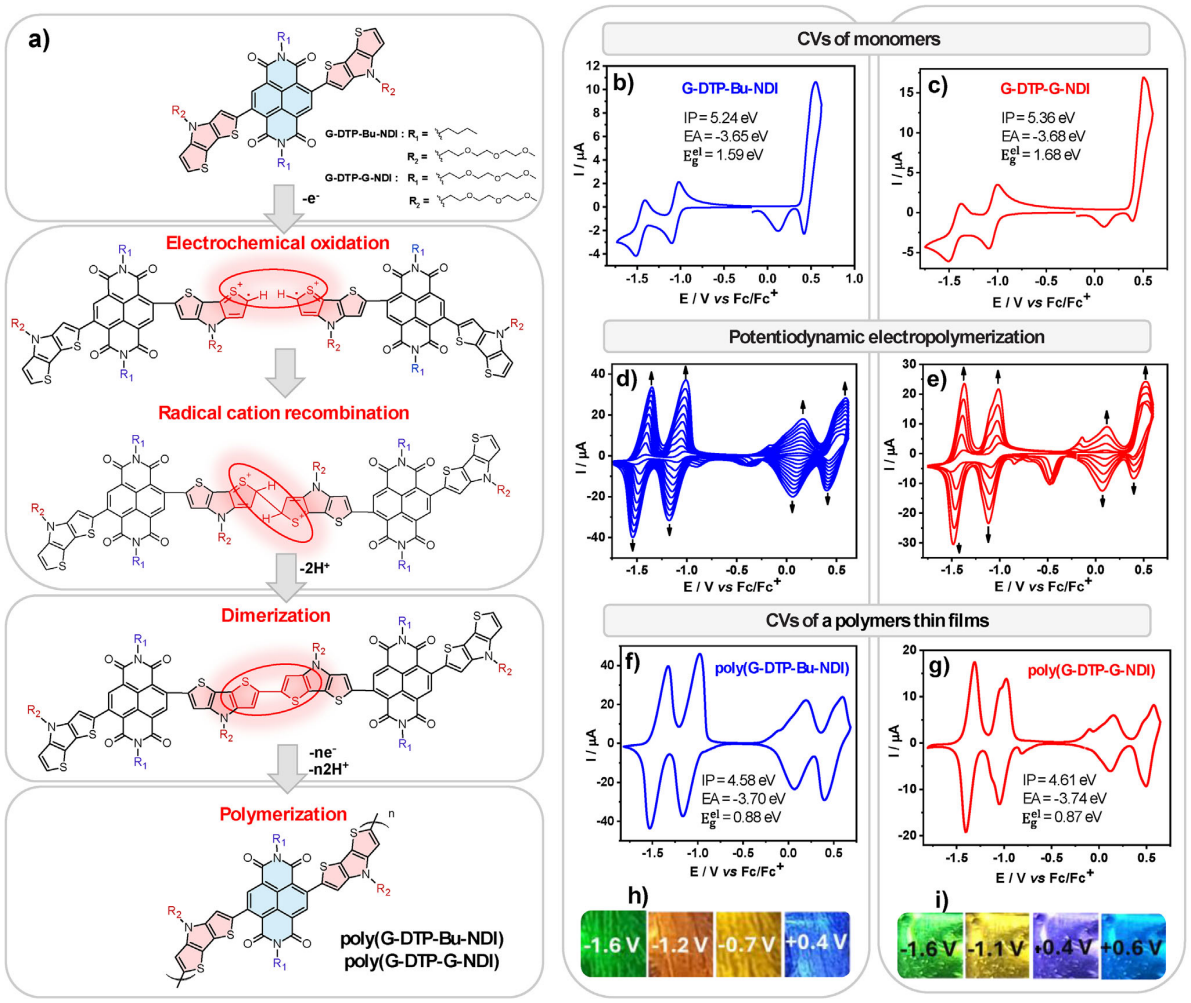
a) Polymerization mechanism of DTP‐NDI derivatives. Cyclic voltammograms of b) **G‐DTP‐Bu‐NDI** and c) **G‐DTP‐G‐NDI** and their potentiodynamic electropolymerization (d, e) on a platinum disc electrode immersed in 0.1 m Bu_4_NPF_6_/DCM electrolyte containing 1×10^−3^ m of the monomer. Cyclic voltammograms of a thin film of f) **poly(G‐DTP‐Bu‐NDI)** and g) **poly(G‐DTP‐G‐NDI)** potentiodynamically deposited on a platinum plate electrode, shown together with photos (h,i) illustrating their color changes determined at different oxidation and reduction states. Electrolyte 0.1 m Bu_4_NPF_6_ in DCM. Scan rates for all registered CVs: 50 mV s^−1^.

The electrochemical properties of the synthesized NDI derivatives were investigated using cyclic voltammetry. The recorded voltammograms are presented in **Figure** **2**b,c. In both cases, in the negative potential range (reported relative to Fc/Fc^+^), two reversible redox couples are observed, corresponding to two consecutive one‐electron reductions of the NDI core to a radical anion in the first step and a spinless dianion in the second one.^[^ ^26^ ^]^ In the positive potential range, for both **G‐DTP‐Bu‐NDI** and **G‐DTP‐G‐NDI**, an anodic peak is observed without a corresponding cathodic peak associated with their irreversible electropolymerization. The electropolymerization will be described in greater detail in the next section of this paper. Table S1 (Supporting Information) presents the values of reduction and oxidation potentials determined from the cyclic voltammetric curves, as well as the values of ionization potentials (IP) and electron affinities (EA) together with the electrochemical bandgap ($E_{g}^{\mathrm{el}})$ calculated based on these electrochemical data.^[^ ^49^ ^]^ As follows from these results, **G‐DTP‐G‐NDI** is slightly easier to reduce and oxidize than **G‐DTP‐Bu‐NDI**, which is reflected in the differences of their |EA| and IP values. Moreover, the IP values are higher than those determined for **EtHex(DTP‐NDI)**, i.e., the already mentioned compound of the same conjugated core but containing alkyl substituents instead of oxyethylene ones. This difference is 0.27 eV^[^ ^26^ ^]^ and most likely indicates that the more flexible glycol chain, compared to the aliphatic 2‐ethylhexyl chain, attached to the donor group DTP, forces a greater twist relative to the plane of the NDI core, thereby increasing orthogonality, which, in turn, results in weakening of the D‐A interactions in the molecule. It is worth noting that the electrochemically determined EA values for all three molecules, regardless of the glycol chain content in their structure, remain similar. Thus, in both cases, the substituents, without significantly changing the redox properties of the investigated compounds, impart an amphiphilic character to them. At the same time, the |EA| values determined for the investigated compounds are much lower than those reported for naphthalene diimides substituted with a glycol chain (NDI‐G: 0.85 eV)^[^ ^50^ ^]^ and for dibrominated naphthalene diimide gNDI‐Br_2_ (≈0.44 eV).^[^ ^48^ ^]^

### 2.3 Electrochemical and Spectroelectrochemical Properties of the Electropolymerization Products

**G‐DTP‐Bu‐NDI** and **G‐DTP‐G‐NDI** were electrochemically polymerized using the potentiodynamic method (Figure 2d,e). The appearance of new redox couples growing in intensity upon consecutive cycles clearly evidences the electropolymerization process, signaling that the polymer layer is growing. The polymerization is initiated by electrochemical generation of radical cations on the *C_a_* atom of the heterocyclic dithienopyrrole ring. In the next step, the formation of a *C_α_‐C_α_* inter‐ring bond occurs through the recombination of the generated radical cations with the simultaneous release of two protons.^[^ ^12^ ^]^

Cyclic voltammograms of electropolymerized compounds, i.e., **poly**(**G‐DTP‐Bu‐NDI)** and **poly(G‐DTP‐G‐NDI**), recorded in a monomer‐free electrolyte, are presented in (Figure 2f,g). The studied dithienopyrrole derivatives of naphthalene diimide exhibit electrochemical activity in both negative and positive potential ranges, giving rise to four reversible redox couples. Those in the negative potential range, at potentials of −1.16 and −1.53 V (vs Fc/Fc^+^) for **poly**(**G‐DTP‐Bu‐NDI)**, and −1.04 and −1.40 V (vs Fc/Fc^+^) for **poly(G‐DTP‐G‐NDI**), are characteristic of the aforementioned two consecutive one‐electron reductions of the naphthalene diimide core of the neutral polymer to the poly(radical anion) and poly(dianion) forms.^[^ ^26^ ^]^ In turn, two redox couples in the positive potential range (+0.19 and +0.60 V vs Fc/Fc^+^ for **poly(G‐DTP‐Bu‐NDI)** and at +0.16 and +0.58 V vs Fc/Fc^+^ for **poly(G‐DTP‐G‐NDI)** are attributed to two one‐electron oxidation processes transforming the neutral polymer to its poly(radical cation) and poly(dication) forms. Moreover, both polymer films exhibit electrochromic properties, exhibiting several stable colors at different potentials. In Figure 2h,i, photos of these polymer layers deposited on a Pt‐plate and recorded at various working electrode potentials are presented. The **poly(G‐DTP‐Bu‐NDI)** layer changes color from green (−1.6 V) to orange (−1.2 V), then to yellow (−0.7 V), and finally to blue (+0.4 V) (Figure 2h). Similarly, the **poly(G‐DTP‐G‐NDI)** layer changes color from green (−1.6 V) to yellow (−1.1 V), then to purple (+0.4 V), and finally to blue (+0.6 V) (Figure 2i).

The values of the reduction and oxidation peak potentials, as well as E_red_(onset), E_ox_(onset), and IP and EA values calculated for **poly(G‐DTP‐Bu‐NDI)** and **poly(G‐DTP‐G‐NDI)** from the electrochemical data, are collected in Table S2 (Supporting Information). The donor–acceptor interactions, combined with extended conjugation, make **poly(G‐DTP‐Bu‐NDI)** and **poly(G‐DTP‐G‐NDI)** low‐bandgap copolymers, exhibiting respective IPs of 4.58 and 4.61 eV (determined from the onset of the first oxidation peak) and |EA| of 3.70 and 3.74 eV (determined from the onset of the first reduction peak). The electrochemical bandgaps, equal to 0.88 eV for **poly(G‐DTP‐Bu‐NDI)** and 0.87 eV for **poly(G‐DTP‐G‐NDI)**, are of 0.71 and 0.81 eV narrower than the gaps of their monomers **G‐DTP‐Bu‐NDI** and **G‐DTP‐G‐NDI**, respectively. Moreover, the presence of glycol chains in the molecular structure of both polymers results in an additional reduction of their bandgap as compared to their aliphatic counterpart **EtHex(DTP‐NDI)**, which $E_{g}^{\mathrm{el}}$ is equal to 1.11 eV.^[^ ^26^ ^]^

**Figure 3:**
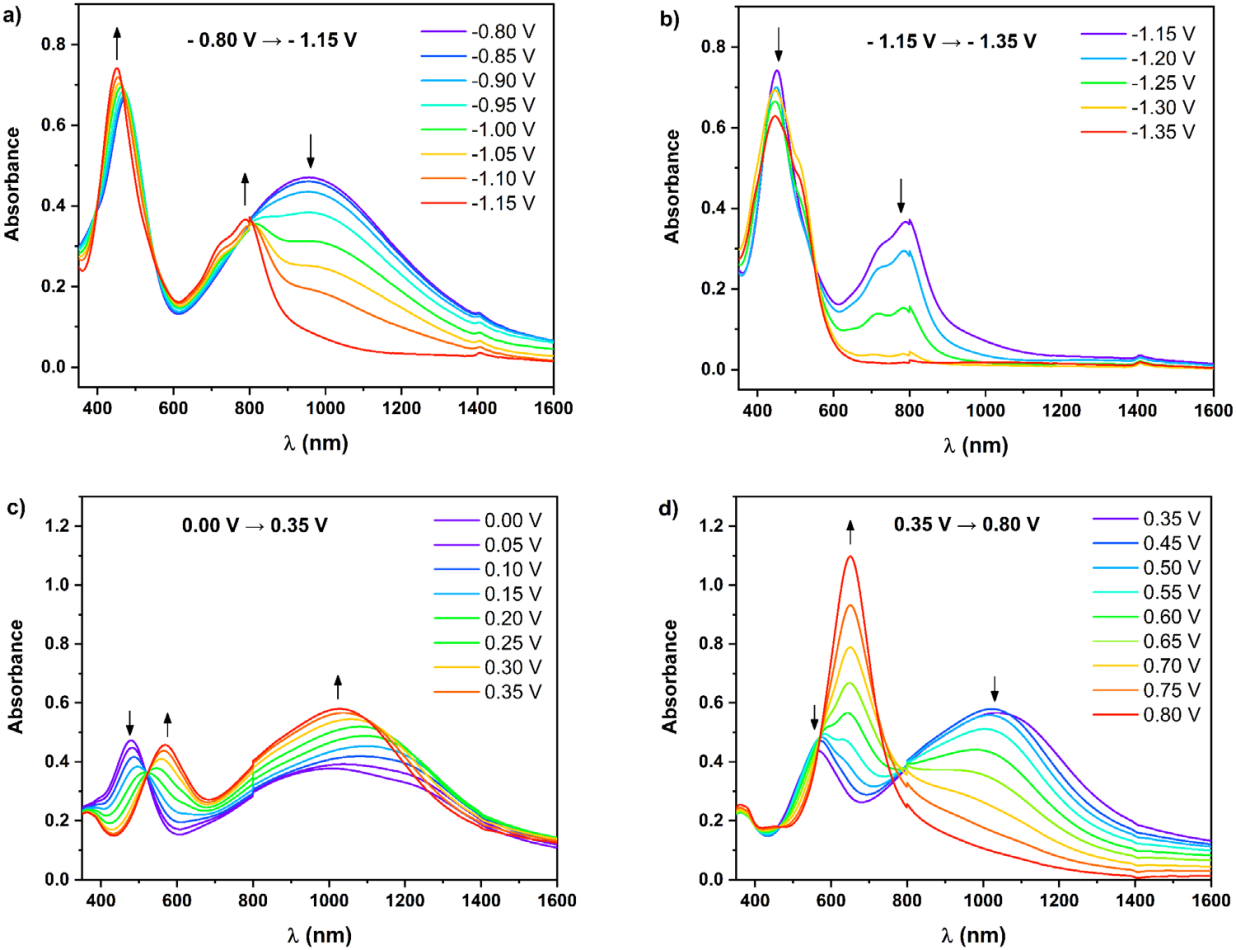
UV–vis–NIR spectra of a thin film of **poly(G‐DTP‐G‐NDI)**, deposited on an ITO electrode and registered for decreasing/increasing electrode potential ranges: a) −0.80 to −1.15 V; b) −1.15 to −1.35 V, c) 0.0 to +0.35 V; b) +0.35 to +0.80 V. Electrolyte 0.1 m Bu_4_NBF_4_/ACN, E versus Fc/Fc^+^.

The spectral evolution induced by the working electrode potential changes was studied in greater detail using spectroelectrochemistry. For this purpose, the polymer layers were deposited on an ITO electrode. The UV–vis–NIR spectra of **poly(G‐DTP‐G‐NDI)** registered at different potentials are presented in **Figure** **3**a,b, whereas those obtained for **poly(Bu‐DTP‐G‐NDI)** can be found in Supporting Information (Figures S13, S14, Supporting Information). Due to significant similarities in the results of these studies, the discussion in the main text will be limited to the case of **poly(G‐DTP‐G‐NDI)**, serving as an example. However, the existing differences will be indicated. Upon reduction of the neutral polymer, first spectral changes appear at E = −0.80 V versus Fc/Fc^+^ and involve a decrease in the intensity of the CT band and a slight increase in the band's intensity at 473 nm (Figure 3a). In the potential range from −0.80 to −1.15 V versus Fc/Fc^+,^ this decrease in the CT band intensity continues, revealing at lower potentials a new band peaked at 801 nm, which reaches its maximum intensity at E = −1.15 V. These changes clearly coincide with the potential range of the first reduction peak in the cyclic voltammogram of **poly(G‐DTP‐G‐NDI)** (compare Figure 3a and Figure 2g). Therefore, the observed spectral changes correspond to the first step of the polymer reduction, transforming its neutral naphthalene diimide units to their radical anion form. The structural changes of **poly(G‐DTP‐(Bu/G)‐NDI)** during p‐ and n‐doping processes are presented in the Supporting Information section (Figure S12, Supporting Information). Upon lowering the potential from −1.15 to −1.35 V, the band at 801 nm, diagnostic of the radical anion state, diminishes in intensity, being totally bleached at E = −1.35 V. Simultaneously, a slight hypsochromic shift of the high‐energy band to 446 nm is observed (Figure 3b). Again, these spectral changes coincide with the potential range of the second reduction peak in the cyclic voltammogram of the polymer (compare Figure 3b and Figure 2i), indicating that they should be attributed to the transformation of the naphthalene diimide units from their radical anion state to the fully reduced spinless dianion state. Rather minor spectral changes in the intensity and the position of the band in the vicinity of 470 nm, accompanying the reduction of NDI to its radical anion form, can be attributed to the local character of this process limited only to a part of the NDI core and influencing to a minor the electron density distribution within the whole macromolecule. In the second reduction step, the formation of a spinless dianion is associated with a rearrangement of the π‐bond sequence but also in the NDI core only.

UV–vis–NIR spectroelectrochemistry reveals higher electrochemical stability of **poly(G‐DTP‐G‐NDI)** than **poly(G‐DTP‐Bu‐NDI)**. In the case of the latter, lowering the potential to the values characteristic of the second reduction peak leads to some degradation processes, which make the registration of the UV–vis–NIR spectrum at potentials inferior to −1.15 V impossible.

Hence, it can be inferred that replacing the butyl groups with glycol chains at NDI likely facilitate charge injection into the layer, thereby reducing its susceptibility to degradation. However, it should be noted that both investigated glycol polymers still exhibit lower electrochemical stability in the second reduction step as compared to their all‐alkylated analogues.^[^ ^24^, ^25^, ^26^ ^]^ The UV–vis–NIR spectral changes of **poly(G‐DTP‐G‐NDI)** induced by the first and second oxidation processes are compared in Figure 3c. In the first step, an increase in the CT band absorbance is observed within the potential range from 0.0 to +0.35 V. Simultaneously, a new band appears with a maximum of 565 nm, while the band at 473 nm diminishes. In the second oxidation step, within the potential range from +0.35 to +0.80 V, a new band emerges with a maximum of 650 nm. Meanwhile, the previously dominant bands fade away (Figure 3d). The isosbestic points formed in both oxidation steps not only indicate the absence of side reactions associated with layer degradation but also demonstrate that the neutral form of the polymer converts into a polycationic one of only one type.

In summary, the two steps of the oxidation process of **poly(G‐DTP‐G‐NDI)** and **poly(G‐DTP‐Bu‐NDI)**, as delineated during the spectroelectrochemical studies, are consistent with their CV characteristics (Figure 2), where the first and second oxidation steps are also distinctly separated. Furthermore, it should be emphasized that the first step of oxidizing the neutral polymer to the radical cation form (referred to as polaron in solid‐state physics) causes a complete change in the sequence of π bonds throughout the macromolecule, not just locally as in the first reduction step. As a result, the polymer spectrum undergoes a complete transformation already in the first oxidation step, becoming typical of a conducting polymer, wherein charge carriers are localized polarons (a specific type of radical cations).^[^ ^51^ ^]^ The transformation of localized polarons into localized bipolarons (spinless dications) in the second oxidation step causes a hypsochromic shift of the lowest‐energy band, similar to the case of other conducting polymers.^[^ ^51^ ^]^ Similar behavior in the positive potential range is also found for **poly(G‐DTP‐Bu‐NDI)** (Figure S14, Supporting Information) and the fully aliphatic polymer analogue of both compounds.^[^ ^26^ ^]^ Positions of the bands which are diagnostic of the neutral, radical anion, and dianion states of **poly(G‐DTP‐Bu‐NDI)** and **poly(G‐DTP‐G‐NDI)** are listed in Table S3 (Supporting Information).

### 2.4 Electrochemical and Spectroscopic Properties in Aqueous Electrolytes

**Figure 4:**
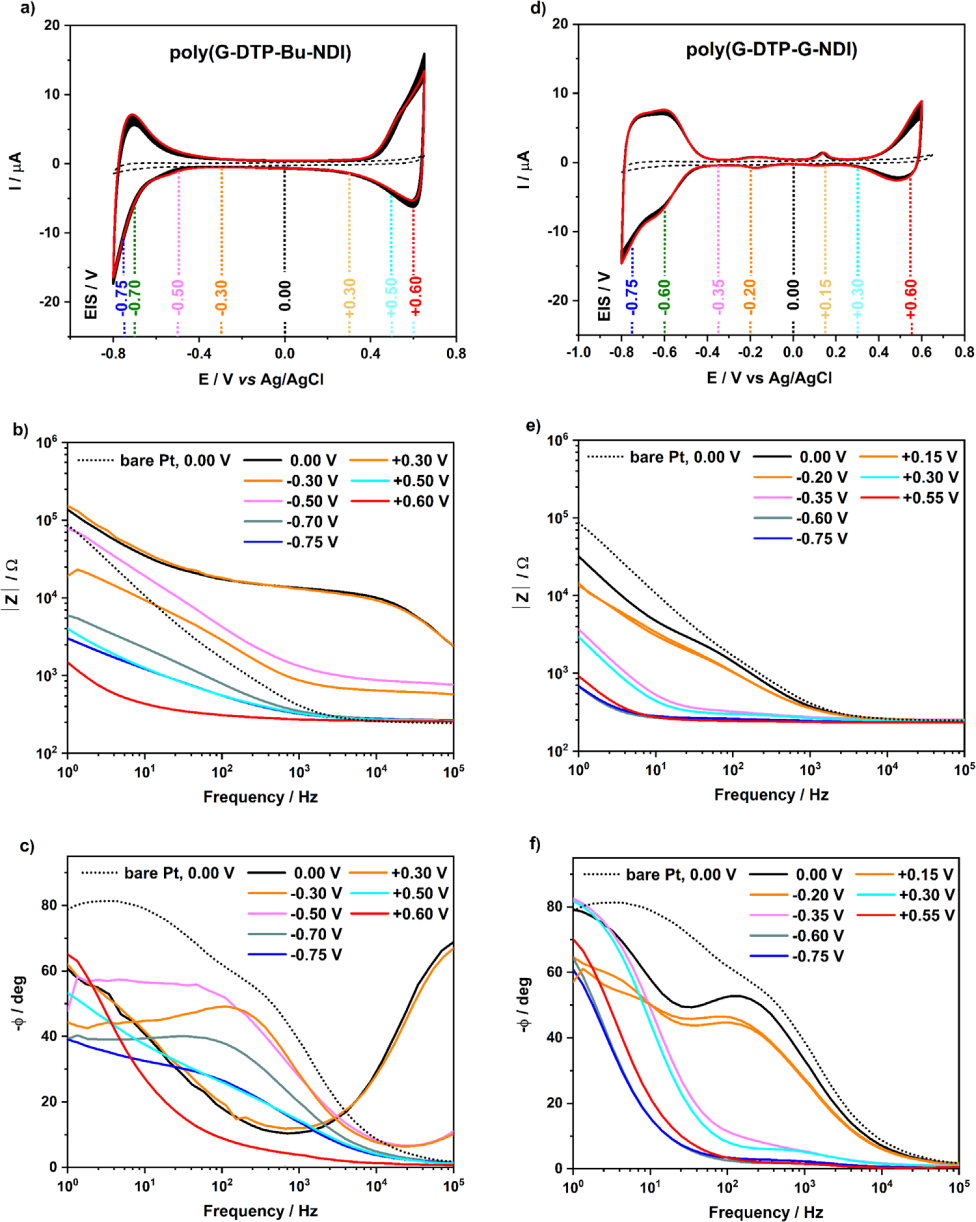
CV and EIS (Bode plots and phase angle changes) measurements of a–c) **poly(G‐DTP‐Bu‐NDI)** and d–f) **poly(G‐DTP‐G‐NDI)** potentiodynamically film‐coated platinum electrodes in 0.1 m NaCl aqueous electrolyte (E vs Ag/AgCl). The CV curves were recorded with a scan rate of 50 mV s^−1^ for 20 and 50 cycles for **poly(G‐DTP‐Bu‐NDI)** and **poly(G‐DTP‐G‐NDI)**, respectively (a red line indicates 1st scan). Dashed black traces in all panels are for a bare Pt electrode, to provide a point of reference for each measurement.

Considering the intended application of the discussed polymers in electrochemical transistors, a detailed investigation of their spectroscopic and electrochemical properties in deaerated aqueous electrolytes was undertaken. The electrochemical responses of thin polymer films deposited on a Pt working electrode by electropolymerization under potentiodynamic conditions were recorded in a three‐electrode configuration in a 0.1 m NaCl aqueous electrolyte (**Figure** **4**). Both polymers reveal electrochemical activity in both the positive and negative potential ranges. Their cyclic voltammograms are conspicuously broad and devoid of discernible features, a hallmark often encountered in films of conjugated polymers characterized by a relatively disordered microstructure.^[^ ^52^ ^]^ Such behavior is typically associated with volumetric charging and discharging processes. However, in the case of **poly(G‐DTP‐G‐NDI)**, a slight indication of the formation of the double reduced state shape is observed. Additionally, the reduction onset value for this polymer is ≈0.18 V, which shifted toward more positive potentials than that of **poly(G‐DTP‐Bu‐NDI)**. This phenomenon appears characteristic of polymer systems with enhanced hydrophilicity, particularly those featuring exclusively glycol side chains.^[^ ^35^ ^]^

Therefore, we studied the electrochemical stability of both polymers concerning potential multi‐cycling under CV conditions in the potential range of −0.8 to +0.65 V versus Ag/AgCl, with a scan rate of 50 mV s^−1^. The measurements show that the polymers are stable over 20 cycles in the case of **poly(G‐DTP‐Bu‐NDI)** and 50 cycles for **poly(G‐ DTP‐G‐NDI)** doping and de‐doping processes. As shown in Figure 4a, upon continuous cycling, the thin film of **poly(G‐DTP‐Bu‐NDI)** exhibits a decrease in electrochemical stability, evidenced by a 5.1% drop in the integrated charge after 20 scans. Similarly, a decrease in electrochemical activity is observed for the **poly(G‐DTP‐G‐NDI)** film, i.e., 4.1% after 20 scans. The electrochemical stability of glycolated polymers depends on the ratio of alkyl substituents to ethyloxy ones. In the case of partly alkylated polymer (**poly(G‐DTP‐Bu‐NDI)**), the electrochemical stability is slightly lower, which is most likely related to the lower tendency of this polymer to swell in water.

Electrochemical impedance spectroscopy (EIS) in aqueous solution is a convenient measurement to establish the suitability of a given material for ECT applications. By applying DC bias during EIS measurement, the polymer can be p‐ or n‐doped, and eventual changes in its conductivity will manifest themselves in changes in the EIS spectrum. An ambipolar material, for instance, would be expected to show a drop in impedance when applying either positive or negative DC bias. Figure 4b,e depicts the complex impedance plots registered for films of **poly(G‐DTP‐Bu‐NDI)** and **poly(G‐DTP‐G‐NDI)** deposited on Pt electrodes. The impedance spectra analysis reveals that high impedance values are observed solely for both polymers' neutral (undoped) forms. Most significantly, the impedance values decrease substantially during these polymers' doping processes (oxidizing or reducing). These changes are particularly pronounced for **poly(G‐DTP‐G‐NDI)**. The impedance of the polymer doped forms reaches significantly lower values (up to two orders of magnitude) than that of the undoped polymer (see spectrum recorded at E = 0.0 V). Figure 4b,e also illustrates plots of the frequency‐dependent phase angle changes of the polymer layers recorded for increasing and decreasing working electrode potential. Analysis of the phase angle spectrum enables us to verify whether the semiconductor in the transistor channel exhibits a more resistive nature (0° < *ϕ* < −45°) or a more capacitive behavior (−45° > *ϕ* > −90°).^[^ ^53^ ^]^ For **poly(G‐DTP‐Bu‐NDI)**, the phase angle value increases solely for the non‐doped forms. In contrast, **poly(G‐DTP‐G‐NDI)** demonstrates no increase in *ϕ* across a broad potential range and for the highest frequency values. Even at frequencies of 10^5^ Hz, this polymer maintains a capacitive character in its neutral form.

**Figure 5:**
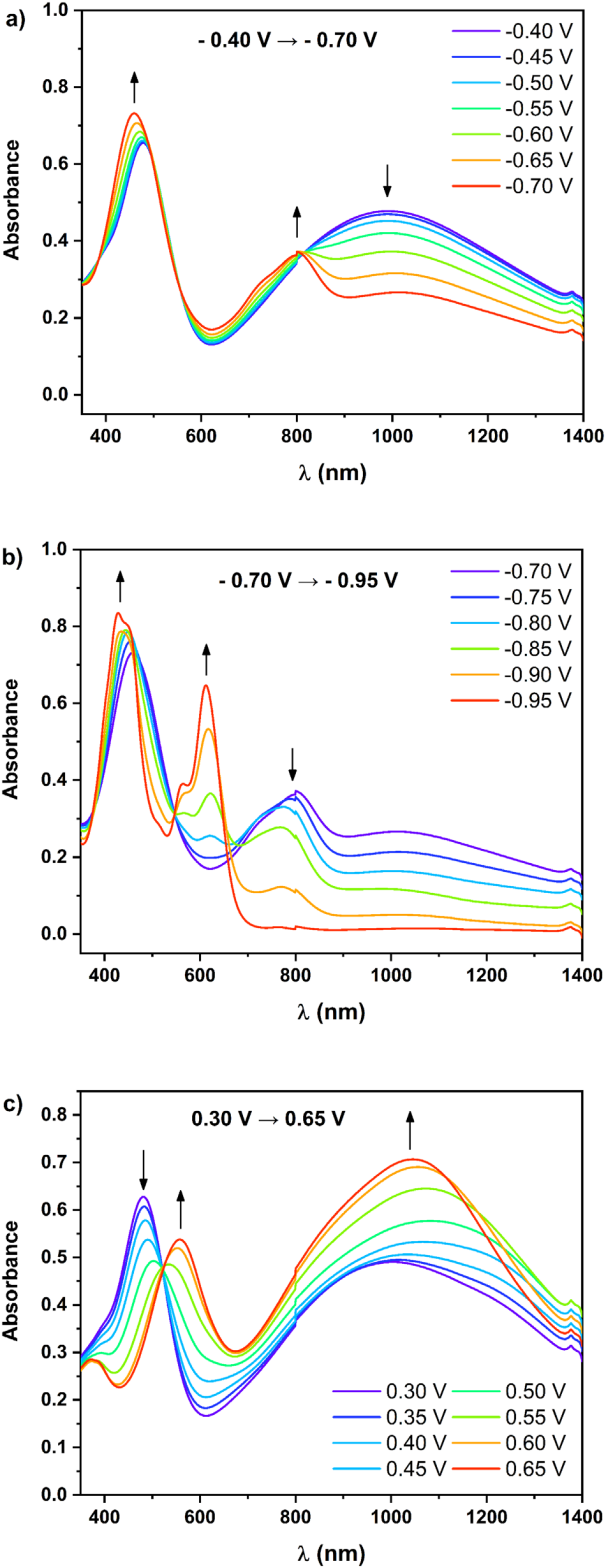
UV–vis–NIR spectra of thin films of **poly(G‐DTP‐G‐NDI)** electrochemically deposited on an ITO substrate, recorded in 0.1 m NaCl aqueous electrolytes for a,b) decreasing and c) increasing electrode potential versus Ag/AgCl.

Spectroelectrochemical analyses were conducted to delve deeper into the p‐ and n‐type doping mechanisms of the electrodeposited polymers in aqueous solution (**Figure** **5**). During the reduction of **poly(G‐DTP‐G‐NDI)** within the potential range of −0.40 to −0.70 V versus Ag/AgCl, a decline in the intensity of the CT band is observed. Concomitantly, a new band peaked at ca. 800 nm is revealed, clearly visible at E = −0.70 V. The high energy band undergoes a hypsochromic shift from 478 to 460 nm, was observed. Furthermore, a new band emerged at 800 nm (Figure 5a). These alterations are attributed to generating a radical anion on the NDI core. Intriguingly, this spectroelectrochemical response of the polymer in an aqueous medium closely parallels that recorded in a non‐aqueous medium (acetonitrile). Assuming the potential of the Fc/Fc^+^ redox pair relative to the Ag/AgCl/0.1 m NaCl electrode is +0.336 V,^[^ ^54^ ^]^ it can be inferred that the potential ranges associated with the observed alterations in both aqueous and non‐aqueous environments are strikingly similar. Upon decreasing the applied potential values to those corresponding to the second reduction step (from −0.75 to −0.95 V vs Ag/AgCl), a new peak at 612 nm and forming an isosbestic point is observed. This indicates that the radical anion form of the polymer transforms into a more reduced dianion form without side products (Figure 5b). Spectral changes in the UV–vis–NIR range observed during the oxidation of the compound in an aqueous environment within the potential range from +0.30 to +0.65 V versus Ag/AgCl closely resemble those observed during the first oxidation step of this compound in non‐aqueous environments (compare Figure 5c and Figure 3c). Similar to the reduction process, considering the potential of the Fc/Fc^+^ redox couple relative to Ag/AgCl/0.1 NaCl (+0.336 V^[^ ^54^ ^]^) yields a comparable range of potential for spectral changes. However, it is noteworthy that in the case of spectroelectrochemical measurements in aqueous solution, the second oxidation step for both examined polymers could not be registered – most likely due to the degradation of their layers combined with the release of oxygen generated by water electrolysis under these conditions (Figures S15, S16, Supporting Information). Additionally, in the case of the less wettable layer of **poly(G‐DTP‐Bu‐NDI)**, only the first step of the oxidation process could be observed (Figure S16, Supporting Information).

### 2.5 OECTs Fabrication and Characteristics

**Figure 6:**
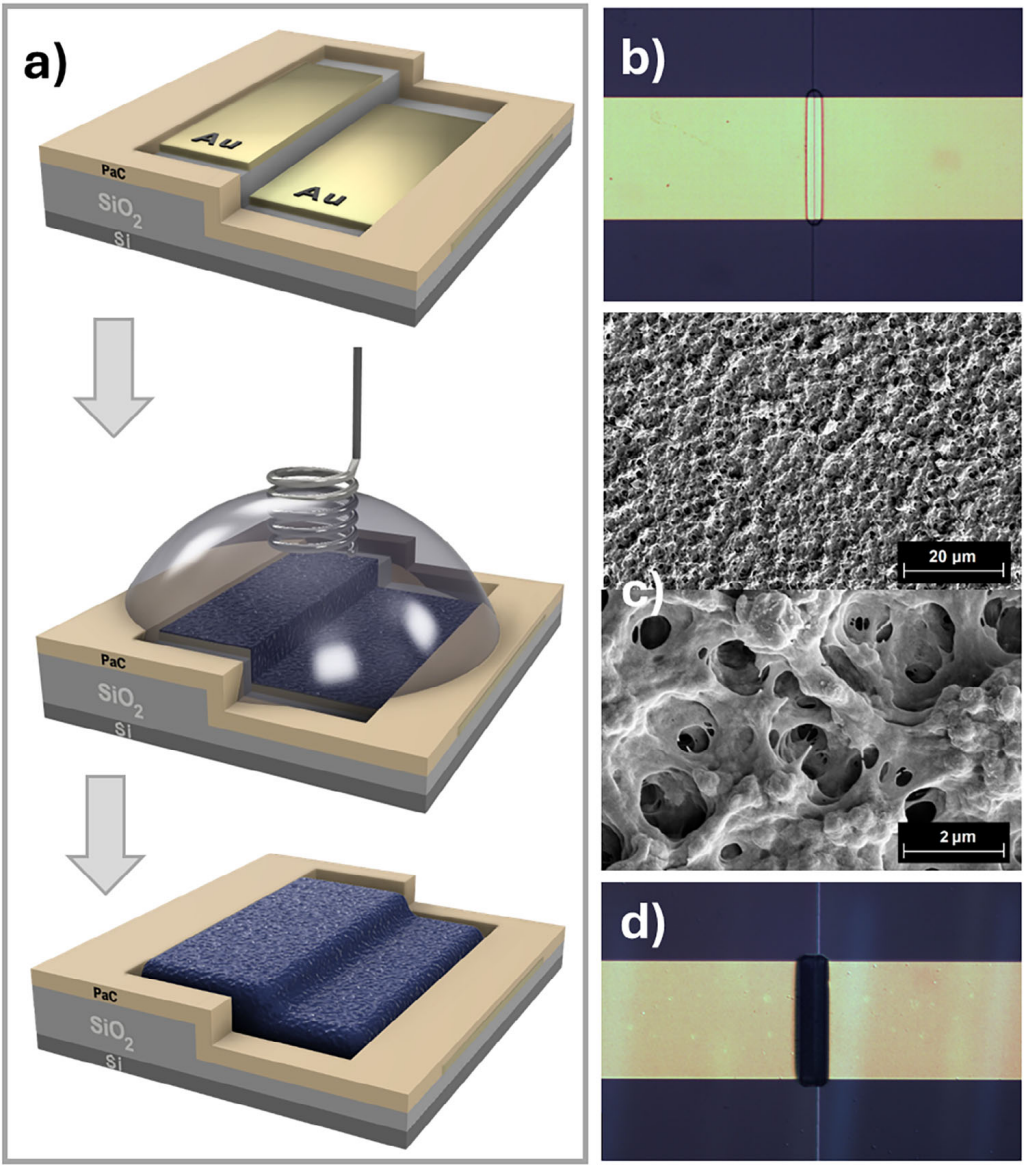
a) A schematic of the in situ electropolymerization process, starting from the vOECT substrate. b) Optical photomicrograph of a *v*OECT substrate (W = 100 µm) before electropolymerization. c) SEM images of the electropolymerized **poly(G‐DTP‐G‐NDI)** channel. d) Optical photomicrograph of the **G‐DTP‐G‐NDI** channel produced with an electropolymerization charge of 2.25 µC.

**Figure 7:**
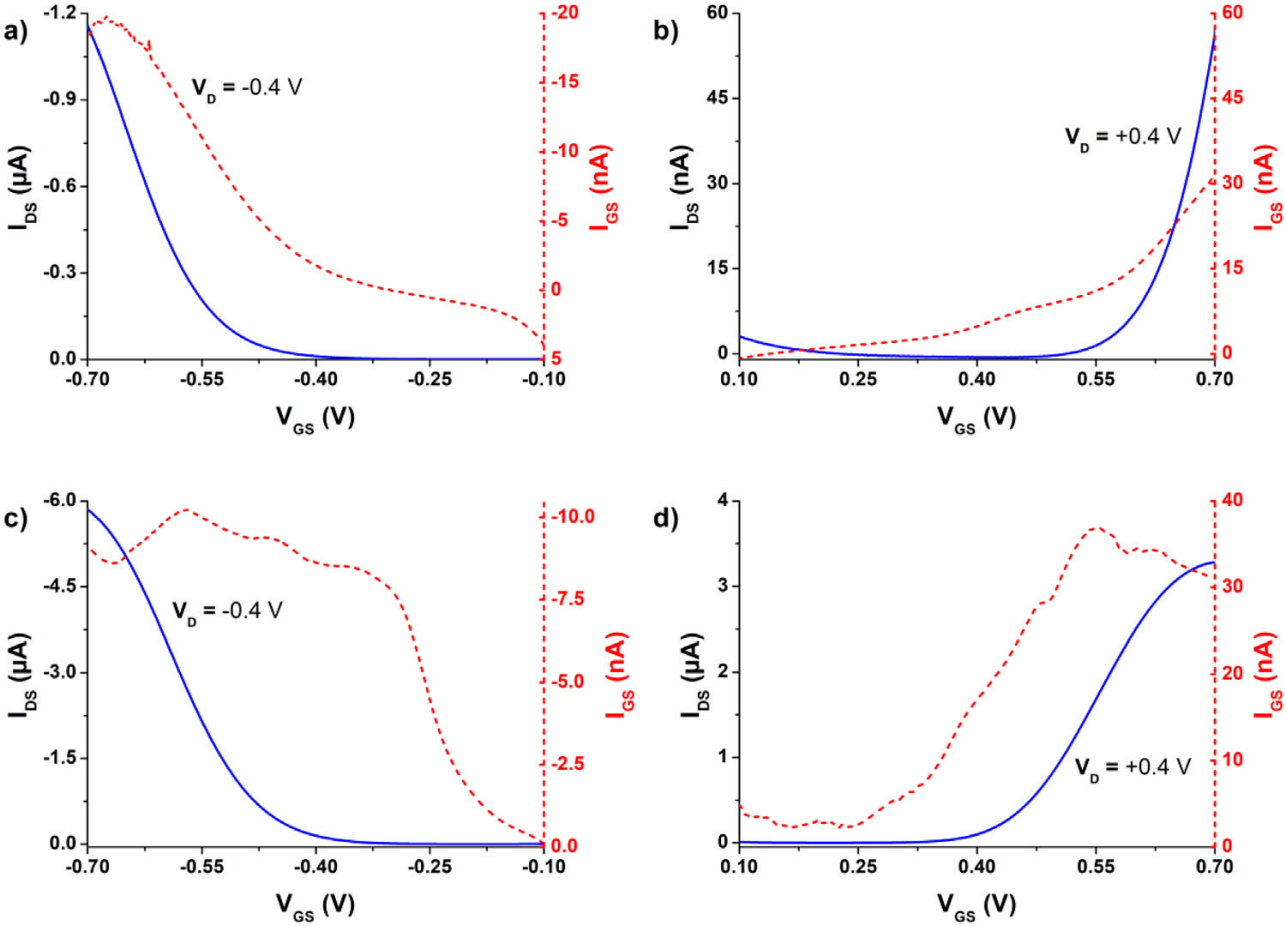
Selected transfer characteristics along with corresponding gate currents for a,b) **poly(G‐DTP‐Bu‐NDI)** or c,d) **poly(G‐DTP‐G‐NDI)** transistors (obtained by electropolymerization with 2.25 µC charge) in (a,c) p‐type and (b,d) n‐type operation. W/L = 100/0.350 µm.

In the concluding part of our work, following the encouraging outcomes obtained from electrochemical and spectroelectrochemical assessments, we studied the behavior and stability of our polymers as channel materials in organic electrochemical transistors operating in an aqueous KCl electrolyte. At this juncture, it is important to highlight two innovations we have carried out. First, we have implemented in situ electrochemical deposition of polymer layers within the transistor channel through galvanostatic electropolymerization. This is an innovative approach since conventionally, polymer materials tested in OECT are solely deposited via solution‐based methods, such as spin‐coating or drop‐casting,^[^ ^35^ ^]^ after their chemical synthesis. Our approach simplifies substrate preparation for polymerization while eliminating the need for extensive polymer purification, inefficient fractionation, and challenges related to the solubility and processability of high molecular‐weight fractions. We assert that the in situ electropolymerization of electroactive material within the transistor channel holds great technological potential. Of particular significance, our polymers were tested in electrochemical transistors featuring a specially designed and still highly innovative, very recently reported, vertical configuration, resulting in a short channel of 350 nm^[^ ^55^ ^]^ (**Figure** **6**). This vertical and short‐length channel is favorable for in situ electropolymerization.^[^ ^19^ ^]^ It is worth noting that, in the literature, the predominant configuration remains the horizontal arrangement with channel lengths in excess of 10 micrometers.^[^ ^56^ ^]^ We measured transfer characteristics of OECTs fabricated from **poly(G‐DTP‐Bu‐NDI)** and **poly(G‐DTP‐G‐NDI)** using negative or positive source‐gate potentials, to evaluate n‐type accumulation and p‐type accumulation regimes of ECT operation, respectively. We observed both types of transport, and therefore we can definitively assign the label of ambipolarity to these polymers (**Figure** **7**).

**Figure 8:**
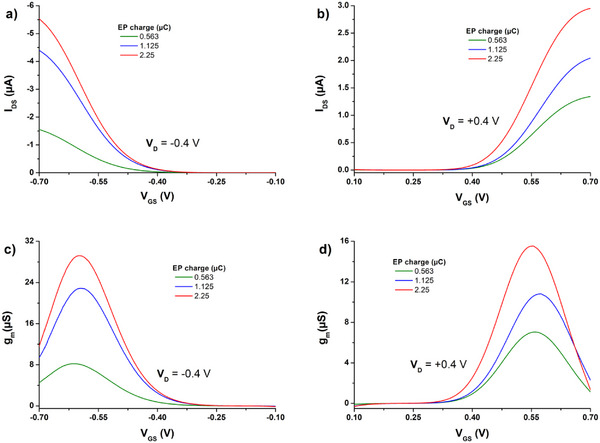
a,b) Transfer and c,d) transconductance curves of **poly(G‐DTP‐G‐NDI)** in (a,c) p‐type and b,d) n‐type operation as a function of different charge passed during electropolymerization (EP). The results presented are averages of 2–3 different samples.

These measured transfer characteristics demonstrate the superior performance of **poly(G‐DTP‐G‐NDI)** compared to **poly(G‐DTP‐Bu‐NDI)**. This is visible as higher peak currents and lower gate currents, especially for n‐type operation. In the case of **poly(G‐DTP‐Bu‐NDI)**, I_GS_ is comparable to or even higher than I_DS_. For instance, in Figure S17b (Supporting Information), part of the n‐type curve cannot be shown in the log scale plot because I_DS_ < 0 for V_GS_ +0.16 to +0.26 V. This indicates that the more extensive side‐chain glycolation makes the backbone more accessible for doping and dedoping and results in better ECT electrochemical switching in **poly(G‐DTP‐G‐NDI)**. Since this material showed more promise in the ECT application, we proceeded to characterize the active layer thickness dependence on ECT performance. In **Figure** **8**, transfer and transconductance characteristics are shown for **poly(G‐DTP‐G‐NDI)** layers of different thicknesses. The thicknesses varied through polymerizations carried out at the same interval (75 s), but the polymerization currents differed and, as a consequence, the polymerization charge. Assuming constant Faraday yield, these procedures should lead to different amounts of deposited polymers in the transistor channel.

**Figure 9:**
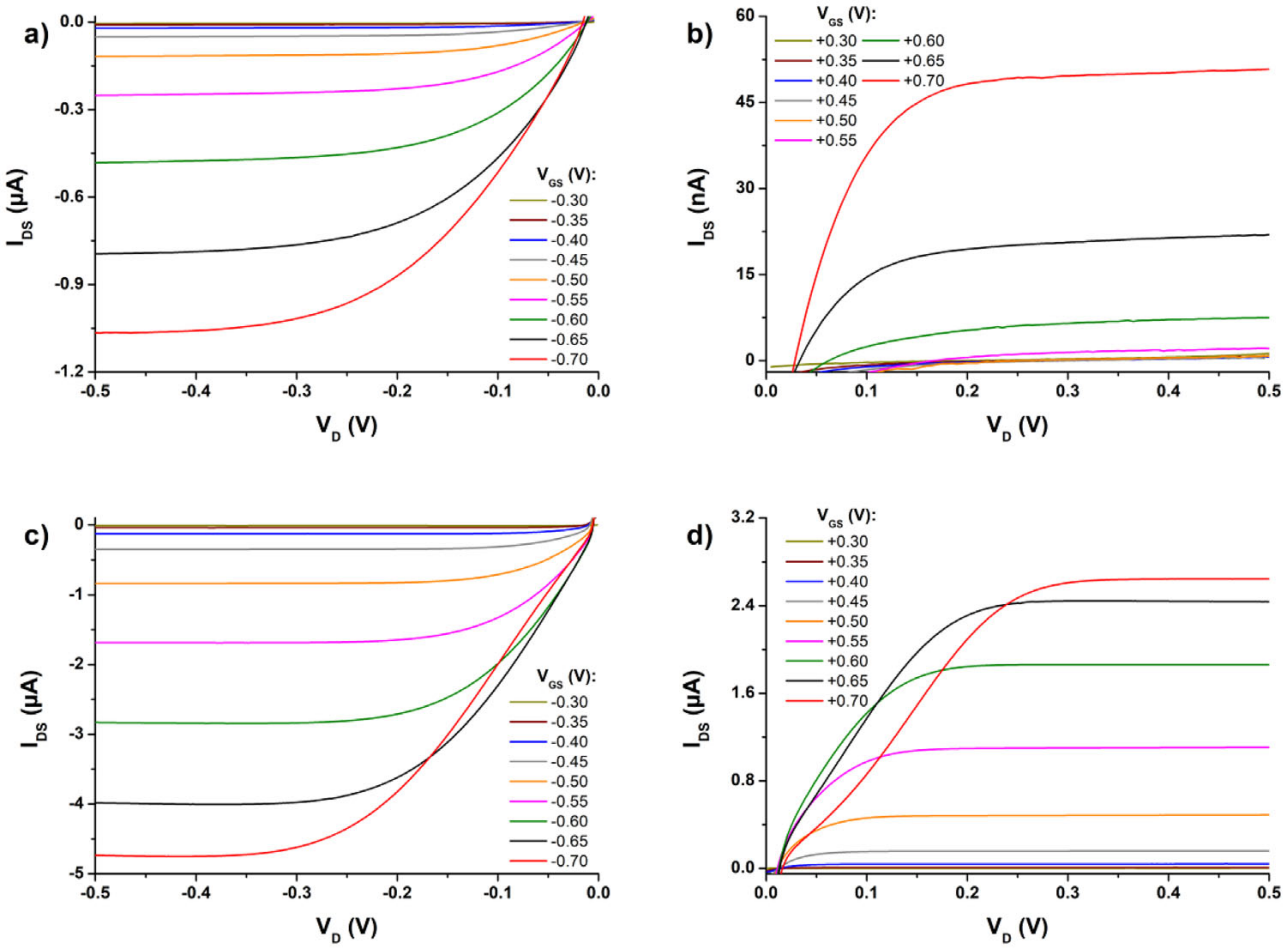
Output characteristics of a,b) **poly(G‐DTP‐Bu‐NDI)** or c,d) **poly(G‐DTP‐G‐NDI)** transistors (obtained by electropolymerization with 2.25 µC charge) in (a,c) p‐type and b,d) n‐type operation.

As expected, the higher the polymerization charge, the higher the I_DS_ values; however, the dependence is not linear. This thickness‐dependent improvement in overall currents as well as transconductance signals that the glycol‐bearing **poly(G‐DTP‐G‐NDI)** is electrochemically‐accessible throughout its volume and thus the volume of the channel can be effectively doped. While transfer characteristics are effective for assessing transconductance and overall doping/dedoping efficiency in ECTs, output characteristics can give us more information about effects like overall conductivity and contact resistance coming from the polymer/SD interfaces. **Figure** **9** presents the output characteristics of transistors with **poly(G‐DTP‐Bu‐NDI)** and **poly(G‐DTP‐G‐NDI)** layers.

**Figure 10:**
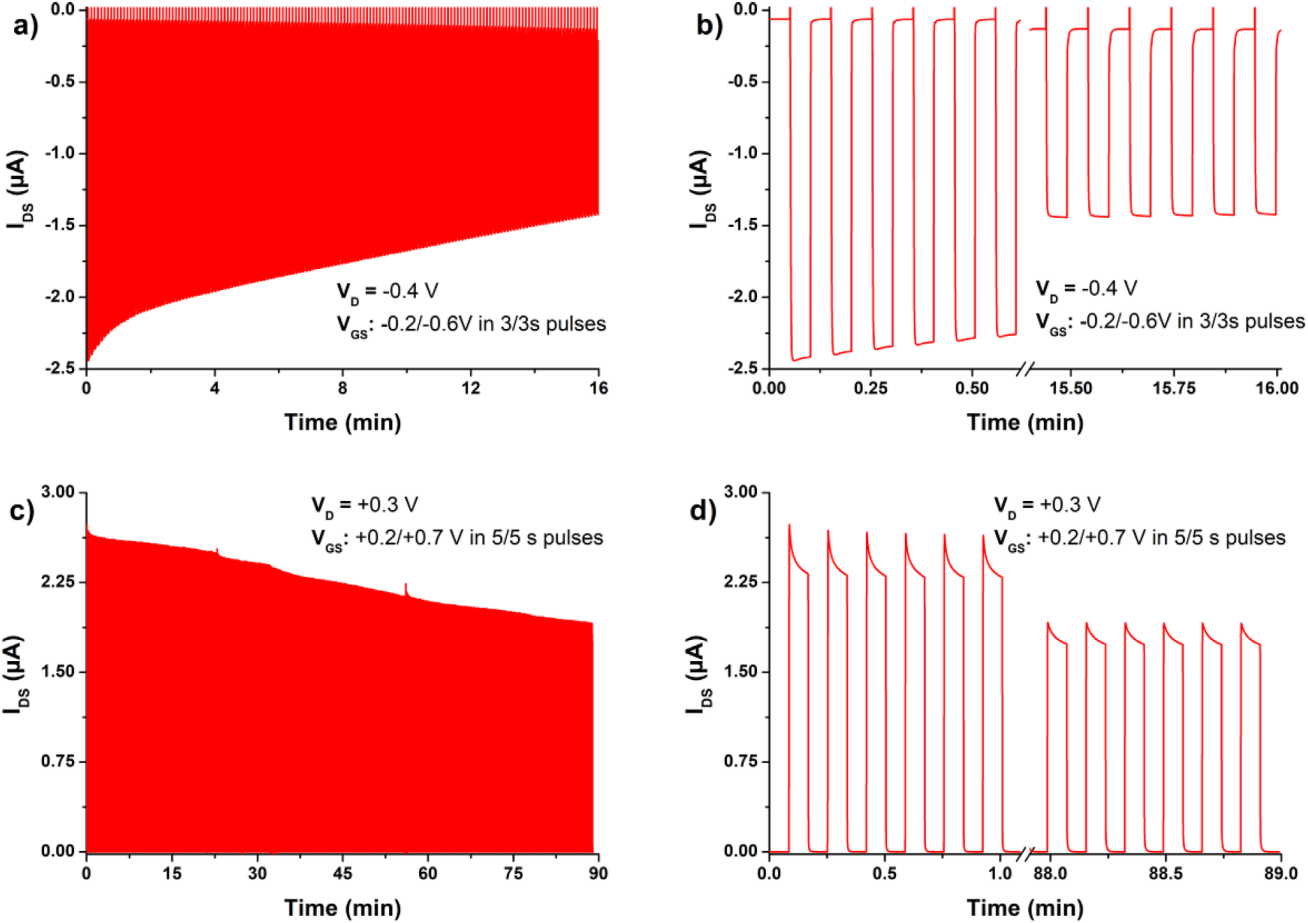
On/off switching stability tests of **poly(G‐DTP‐G‐NDI)** (2.25 µC EP charge) transistors in (a,b) p‐type and (c,d) n‐type operation. Justification of the test parameters (values of V_GS_, V_D_, and the time interval) is given in the supplementary information. For better visualization, full plots (a,c) are supported with break‐type plots (b,d) limited to the initial and terminal phases of the same tests.

As can be seen in the plots, the case of **poly(G‐DTP‐G‐NDI)** curves recorded for high V_GS_ show lower I_DS_ values for low V_D_, especially for n‐type transport, where V_D_ has to be at least +0.25 V to see constantly increasing I_D_ with the increase of V_GS_. This phenomenon, caused by the existence of contact resistance, is already reported in the literature.^[^ ^57^ ^]^ For eventual ECT applications, cyclic stability of active polymer materials is of paramount importance. Therefore we conducted switching tests on the **poly(G‐DTP‐G‐NDI)** transistors (3 s on/ 3 s off) at V_D_ voltages where we were able to see drops in stability. These tests showed a decline over 160 cycles of p‐type operation and 500 cycles of n‐type (**Figure** **10**). Overall n‐type operation was more resistant to degradation, signaling a likely irreversible oxidation reaction in the polymer, or perhaps oxidative degradation of the Au underlying contact in the chloride‐containing electrolyte. While this performance decline is problematic for some applications, we would note that it leaves optimism as few ambipolar ECT materials interrogate stability at all. Future research should resolve which part of the device is responsible for this performance drop—the polymer itself, or one or more of the metal/polymer interfaces.

The increased content of glycol side chains in poly(G‐DTP‐G‐NDI) had only a minor effect on the redox properties of the material in the organic electrolyte, compared to the polymer with an aliphatic butyl chain at the naphthalenediimide acceptor unit, poly(G‐DTP‐Bu‐NDI). However, our findings confirm that in aqueous electrolytes, a higher content of glycol side chains in poly(G‐DTP‐G‐NDI) significantly improves ion‐electron coupling, resulting in greater stability and higher efficiency in OECT operation. First, the enhanced ionic conductivity in OECTs with an active layer of poly(G‐NDI‐G‐DTP) facilitates more efficient ion transport, leading to the generation of higher drain currents. Moreover, these transistors exhibit faster current response, requiring a lower gate voltage to effectively open the transistor channel, thereby improving switching efficiency. This behavior can be attributed to the increased swelling capacity of poly(G‐DTP‐G‐NDI) in aqueous electrolytes, which is critical for achieving stable and reversible electrochemical operation. The efficient penetration of electrolyte ions into the bulk material is directly linked to the high content of glycol groups in the polymer chain. In turn, uniform ion penetration and reversible doping contribute to the high operational stability of OECTs. Conversely, in polymers with low contents of glycol substituents, such as poly(G‐DTP‐Bu‐NDI), the tendency to swell is significantly reduced, restricting ion diffusion into the polymer matrix. This leads to heterogeneous doping and dedoping, which results in localized charge accumulation and increased mechanical stress, ultimately causing structural degradation. As a consequence, the (de)doping process becomes less reversible, and the electrochemical stability of the polymer is compromised. Hence, fine‐tuning the balance between mechanical and electrochemical stability in OECT materials is crucial for designing new electroactive materials that enhance both device performance and long‐term stability.

## 3 Conclusion

This study presented the synthesis, comprehensive characterization, and successful application of novel glycolated dithienopyrrole‐naphthalene diimide based copolymers, **poly(G‐DTP‐Bu‐NDI)** and **poly(G‐DTP‐G‐NDI)**, within the domain of vertical organic electrochemical transistors—*v*OECT. The integration of glycolated side chains imposed an amphiphilic nature on the polymers and thus enhanced their electrochemical properties in aqueous electrolytes. Through spectroscopic and electrochemical assessments, both copolymers exhibited significant ambipolarity and stability across multiple electrochemical scans, proving their robustness in both aqueous and organic environments. Particularly notable is their ability to form thin, porous, uniform electroactive layers through in situ electropolymerization directly within the OECT channel, bypassing traditional film deposition methods, which are often cumbersome, less precise, and wasteful in their materials usage. An undeniable advantage of electropolymerization is the absence of palladium contamination in the active layer, which is a recognized problem in the field of OECTs. Moreover, electropolymerization allows the deployment of copolymers that would be difficult or impossible to synthesize and purify via conventional solution‐chemistry means. The advantage of copolymers is the ability to craft a final material with ambipolar electrochemistry and charge transport. The electrochemical analyses, starting from EIS as a function of DC bias, confirmed that these materials support both n‐type and p‐type channel operations, showcasing their versatility and making them prime candidates for advanced sensing applications, especially in biologically relevant environments. Their distinct electrochromic properties, demonstrated through a broad range of color changes under various applied potentials, further underline their utility in real‐time sensing applications where visual monitoring of processes is advantageous. Looking forward, the promising attributes of **poly(G‐DTP‐Bu‐NDI)** and **poly(G‐DTP‐G‐NDI)** emphasize the potential of donor–acceptor copolymers in the field of bioelectronics, particularly for the development of next‐generation biosensors and interfaces with living tissues. Ongoing improvements in polymer chemistry and device engineering are expected to expand the capabilities of OECTs further, enabling more sensitive, stable, and multifunctional bioelectronic devices. Integrating such advanced materials into OECTs paves the way for innovative applications in biomedical engineering, environmental monitoring, and beyond, reinforcing the critical role of material science in the evolution of organic electronics. What remains to be improved in the field of ambipolar polymers is operational stability. From CV scans, it is clear that there is instability related to irreversible oxidation reactions, which correlates to poorer stability of p‐type ECT operation versus more stable n‐type operation. It will be necessary to use molecular engineering to passivate weak points where oxidative degradation can begin in these types of polymers, as we, and others, find that cyclic redox reactions on these polymers lead to decreases in conductivity.

## 4 Experimental Section

The supporting information details the monomer synthesis and characterizations, UV–vis spectra, spectroelectrochemistry, and OECT measurements.

## Conflict of Interest

The authors declare no conflict of interest.

## Supporting information



Supporting Information

## Data Availability

The data that support the findings of this study are available in the supplementary material of this article.
