# Supplementary material for: In Situ Electropolymerized Ambipolar Copolymers for Vertical OECTs
Source: Small. 2025 Apr 30;21(30):2411219. doi: 10.1002/smll.202411219 (PMC12306382; doi:10.1002/smll.202411219)
Supplement: Supplementary file 1 — Supporting Information [file SMLL-21-2411219-s001.pdf]

NANO · MICRO
small

Supporting Information

for *Small*, DOI 10.1002/smll.202411219

In Situ Electropolymerized Ambipolar Copolymers for Vertical OECTs

Roman Gańczarczyk, Magdalena Rudowska, Maciej Gryszel, Adam Proń, Renata Rybakiewicz-Sekita and Eric D. Głowacki**

Supporting Information

In situ electropolymerized ambipolar copolymers for vertical OECTs

Roman Gańczarczyk[1,2], Magdalena Rudowska[1], Maciej Gryszel[3], Adam Proń[1], Renata Rybakiewicz-Sekita*[1,4], Eric D. Głowacki*[1,2]

[1] Warsaw University of Technology, Faculty of Chemistry, Noakowskiego 3, 00-664, Warsaw, Poland

[2] Bioelectronics Materials and Devices Laboratory, Central European Institute of Technology, Brno University of Technology, Purkyňova 123, Brno 61200, Czech Republic

[3] Linköping University, Laboratory of Organic Electronics, ITN, Bredgatan 33, 60 174 Norrköping, Sweden, e-mail: eric.daniel.glowacki@ceitec.vutbr.cz

[4] Cardinal Stefan Wyszynski University, Faculty of Mathematics and Natural Sciences, School of Exact Sciences, Woycickiego 1/3, 01-938, Warsaw, Poland, e-mail: r.rybakiewicz@uksw.edu.pl

1. Materials and Characterization techniques……………………………………………..S2
2. Synthesis and Characterization…………………………………………………………S4
3. Spectroscopic, electrochemical and spectroelectrochemical studies……………...S21
4. OECTs fabrication characteristics………………………………………………………...S26
5. References……………………………………………………………………………....S26

1. Materials and Characterization techniques

1.1. Materials

Diisopropyl azodicarboxylate 98%, *p*-toluene sulfonyl chloride, sodium chloride, tributyltin chloride 96%, 1,2-dimethylethylenediamine, phthalimide, tetrabutylammonium hexafluorophosphate, copper iodide, hydrazine monohydrate, *n*-butylamine, *n*-butyllithium (2.5 M in hexane), tetrakis(triphenylphosphine)palladium(0) 99%, triphenylphosphine, potassium carbonate, benzamide 99%, tetrabutylammonium hydrogen sulphate (VI) were purchased from Merck. Magnesium sulfate and sodium hydroxide were acquired from Chempur. 2,6-Dibromo-1,4,5,8-naphthalenetetracarboxylic acid dianhydride and triethylene glycol monomethyl ether were obtained from TCI Chemicals. Glacial acetic acid and 3,3'-dibromo-2,2'-bitiophene were received from POCH and Fluorochem, respectively. Dry solvents for all syntheses, solvents for extraction and column chromatography were purchased from Merck, as well as silica gel 60 (0.040 – 0.063 mm) for column chromatography.

1.2. Characterization techniques

^{1}H spectra were recorded on a Bruker spectrometer at 500 MHz or 400 MHz, whereas ^{13}C NMR spectra were recorded on a Bruker spectrometer at 126 MHz or 101 MHz using $CDCl_3$ as the solvent. Mass spectra were measured by ESI method on a Maldi SYNAPT G2-S HDMS mass spectrometer. Absorption spectra of the synthesized compounds dissolved in dichloromethane and those of the polymer thin films deposited onto an ITO electrode were measured using a Varian Cary 5000 spectrophotometer. Cyclic voltammetry experiments were conducted on all synthesized compounds' solutions and thin solid films. A solution containing 1×10^{-3} M G-DTP-Bu-NDI or G-DTP-G-NDI in 0.1 M Bu_4NPF_6 electrolyte dissolved within deoxygenated DCM was introduced into a one-compartment, three-electrode electrochemical cell. This setup comprised a platinum disk working electrode with a surface area of 2 mm^2, a platinum counter electrode, and an Ag/0.1 M Ag^+/acetonitrile reference electrode. Thin polymer films were electrochemically deposited on a platinum disk (for CV and EIS), platinum plate (for electrochromic imaging) or on an ITO (indium–tin oxide) electrode (for UV-vis-NIR spectroelectrochemical investigations. Subsequently, measurements were conducted under nonaqueous (0.1 M Bu_4NBF_6 in DCM or ACN) and aqueous (0.1 M NaCl in water) conditions. The measurements were performed using an Ivium Vertex.One potentiostat. For UV-vis–NIR spectroelectrochemical investigations, the same platinum counter and reference (Ag/0.1 M Ag^+) electrodes were utilized, along with identical electrolytes (0.1 M Bu_4NBF_6/DCM or ACN and 0.1 M NaCl/H_2O) as those employed in the cyclic voltammetry studies. In all electrochemical and spectroelectrochemical measurements in an organic electrolyte, the ferrocene/ferrocenium (Fc/Fc^+) redox couple was used for referencing the obtained data. After each measurement cycle for a given sample, a small amount of ferrocene was added to the electrolytic solution and the redox potential of the Fc/Fc^+ couple was measured. The determined Fc/Fc^+ potential value in dichloromethane with 0.1 M NBu_4PF_6 typically was: 0.175 V *vs* Ag/0.1 M $AgNO_3$, ACN. The determined Fc/Fc^+ potential value in acetonitrile with 0.1 M NBu_4PF_6 typically was: 0.050 V *vs* Ag/0.1 M $AgNO_3$, ACN.

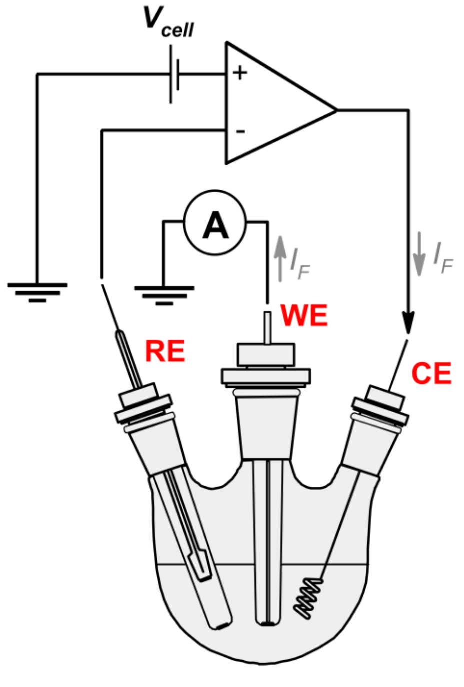

Scheme S1. Schematic of the experimental setup for cyclic voltammetry, impedance spectroscopy, and spectroelectrochemical measurements.

1.3. In situ OECTs channel formation by electrochemical polymerization

Deposition of polymer layers was performed by galvanostatic electropolymerization (2-electrode arrangement) using Ivium Vertex.One potentiostat. The electrolyte (1 mM of a monomer and 100 mM tetrabutylammonium hexafluorophosphate in acetonitrile) was loaded to a modified 1 ml plastic syringe (with a small Pt plate confined inside to serve as a counter electrode) by adjusting the pressure with another syringe connected by flexible tubing. The *v*OECT substrate was precisely positioned under the syringe tip to achieve electrolytic contact without any mechanical connection. The electrical contact to the working electrode terminal was carefully established with two-needle probes placed on both contact pads, allowing simultaneous growth of the material on both source and drain of the *v*OECT. Tetrabutylammonium hexafluorophosphate turned out to be the best electrolyte in terms of transistor performance and reproducibility. We also tested depositions with tetrabutylammonium perchlorate (getting similar results) and $LiClO_4$ and $LiCF_3SO_3$, which resulted in worse-performing transistors.

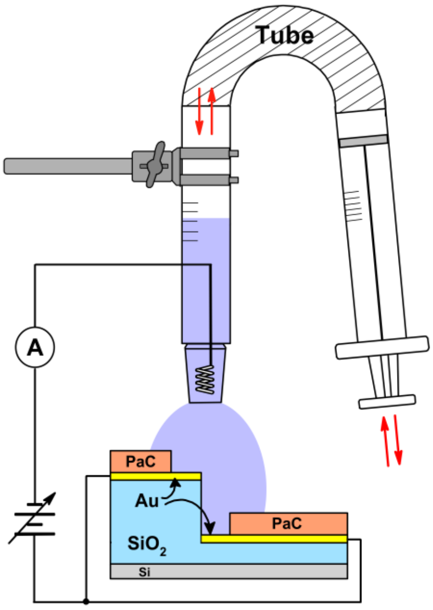

Scheme S2. Schematic of the electropolymerization setup for in situ OECT channel formation.

1.4. Characterization of the electrochemical transistors

All OECT characterizations were performed with an Ag/AgCl pellet as a gate and phosphate buffer saline solution (1x, pH = 7.4). Transfer and output characteristics were recorded with 20 mV/s and 50 mV/s scanning rates, respectively, using a dual channel Keithley K2600A source meter and a custom-made LabView script. Due to the poor performance of **G-NDI-Bu-DTP** transistors, stability tests were performed only for **G-NDI-G-DTP** devices. The p-type stability test was done with a constant V_D value (-0.4 V), alternating V_{GS} between 0 V and -0.6 V in 3/3 s pulses. Initial tests with 0/-0.7 V pulsing showed very poor stability in such conditions. The n-type stability test was performed similarly. Aiming for the highest possible ON/OFF I_{DS} ratio, V_D was set to +0.3 V, V_G was alternated between +0.2 V and +0.7 V. At V_D = +0.4 V and V_G = 0 V, I_{DS} values were relatively high (ap. 5% of IDS at VG = +0.7 V). Using a lower V_D value than recording the transfer curves (+0.4 V) did not result in a significant I_{DS} drop in the ON state. Considering the shape of current pulses, compared to p-type pulsing, V_{GS} switching in an n-type stability test was performed with longer time intervals (5/5 s). SEM images were taken with SEM Thermo Scientific Helios 5 PFIB CXe at an acceleration voltage of 5 kV. The samples were coated with 10 nm of gold before imaging.

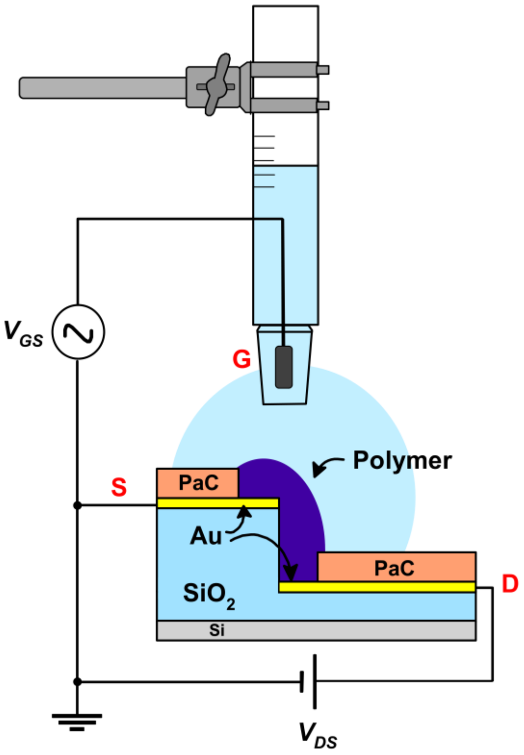

Scheme S3. Schematic of the experimental setup for OECT characterization.

2. Synthesis and Characterization

The synthesis of the 4-(2-(2-(2-methoxyethoxy)ethoxy)ethyl)-2-(tributylstannyl)-4*H*-dithieno[3,2-*b*:2',3'-*d*]pyrrole (G-DTP-$SnBu_3$) commenced with the cyclization of 3,3'-dibromo-2,2'-bithiophene to 4*H*-dithieno[3,2-*b*:2',3'-*d*]pyrrole (H-DTP) according to the modified published procedures[1–3]. 4-(2-(2-(2-methoxyethoxy)ethoxy)ethyl)-4*H*-dithieno[3,2-*b*:2',3'-*d*]pyrrole (G-DTP) was synthesized using Ullmann intramolecular cyclization reaction following a previous report[4]. Subsequently, through Ullmann reaction, 2-(2-(2-methoxyethoxy)ethoxy)ethyl 4-methylbenzenesulfonate (G-Tos) was coupled with H-DTP to obtain 4-(2-(2-(2-methoxyethoxy)ethoxy)ethyl)-4*H*-dithieno[3,2-*b*:2',3'-*d*]pyrrole (G-DTP), which was further subjected to lithiation and stannylation according to literature-known but modified procedures[5]. The synthesis of G-Tos, obtained through a one-step reaction of *p*-toluenesulfonyl chloride with monomethyl ether of triethylene glycol[6], played a crucial role. An important step in the imidization of the dianhydride 2,6-dibromonaphthalene-1,4,5,8-tetracarboxylic acid was the preparation of commercially unavailable 2-(2-(2-methoxyethoxy)ethoxy)ethyl-1-amine (G-NH_2). This compound was prepared in two steps, initially using a Mitsunobu reaction leading to the formation of 2-(2-(2-(2-methoxyethoxy)ethoxy)ethyl)isoindoline-1,3-dione (G-Ftal). The reduction of G-Ftal with hydrazine monohydrate and ethanol at 100°C for 18 hours finally yielded the desired G-NH_2 product. *n*-Butylamine (Bu-NH_2) used for the imidization of Bu-NDI was commercially available. All synthesized compounds were characterized by ^{1}H NMR and ^{13}C NMR spectrometry and, in selected cases, also by MS (ESI) analysis.

2.1. Synthesisi of 2-(2-(2-methoxyethoxy)ethoxy)ethyl 4-methylbenzenesulfonate (G-Tos)

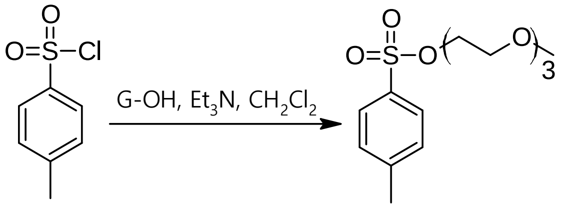

Triethylene glycol monomethyl ether (10.0 g, 60.9 mmol), triethylamine (12.7 ml, 91.4 mmol), and dichloromethane (100 ml) were added to a flask and cooled to 0°C. Then, the solution of *p*-toluene sulfonyl chloride (15.09 g, 79.2 mmol) in 200 ml of dichloromethane was added dropwise, and the reaction mixture was stirred for 12 h at rt. After this time, the solvent was evaporated, and ethyl acetate (30 ml) was added. An obtained precipitate of HCl-Et_3N was drained under reduced pressure and washed with ethyl acetate. The filtrate was extracted with water, 2 M hydrochloric acid solution, saturated sodium bicarbonate solution, and water. The organic phase was dried over anhydrous magnesium sulfate ($MgSO_4$) and concentrated on a rotary evaporator. The crude product was purified by column flash chromatography using a hexane and ethyl acetate solvent mixture (10:1 (*v/v*)). The pure product was a colourless liquid (18.14 g, 94%). **^{1}H NMR** (500 MHz, $CDCl_3$) δ 7.81 – 7.77 (m, 2H), 7.35 – 7.32 (m, 2H), 4.17 – 4.14 (m, 2H), 3.70 – 3.67 (m, 2H), 3.62 – 3.58 (m, 6H), 3.54 – 3.51 (m, 2H), 3.36 (s, 3H), 2.44 (s, 3H). **^{13}C NMR** (126 MHz, $CDCl_3$) δ 144.91, 133.14, 129.94, 128.11, 72.03, 70.87, 70.70, 70.68, 69.36, 68.80, 59.16, 21.77.

2.2. Synthesis of 4*H*-dithieno[3,2-*b*:2',3'-*d*]pyrrole (H-DTP)

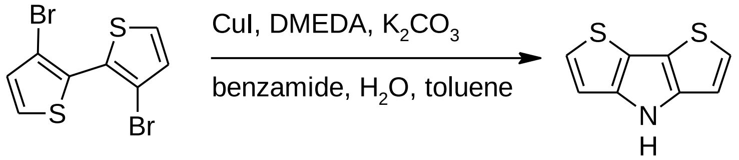

Copper iodide (0.440 g, 2.31 mmol), 1,2-dimethylethylenediamine (0.40 g, 4.63 mmol), and anhydrous toluene (70 ml) were added to a flask and stirrer for 15 minutes under an argon atmosphere. Then 3,3'-dibromo-2,2'-bitiophene (5.00 g, 15.43 mmol) and K_2CO_3 (6.40 g, 46,29 mmol) were added, followed by 5-minute stirring and addition of benzamide (2.24 g, 18.52 mmol) and water (100 µl). The reaction mixture was heated at 110°C for 24 hours. After this time, the precipitate formed was drained off, the filtrate was extracted with dichloromethane and washed with water, then dried over anhydrous magnesium sulfate and concentrated. The crude product was purified by column chromatography using ethyl acetate and hexane mixture (1:9 (*v/v*)). Finally, two fractions were collected: 4*H*-dithino[3,2-*b*:2',3'-*d*]pyrrole (1.26 g) and *N*-benzoyl dithieno[3,2-*b*:2',3'-*d*]pyrrole (1.60 g). The second fraction (1.60 g, 5.65 mmol) was hydrolyzed with methanol, water, chloroform, and potassium carbonate (1.50 g, 10.87 mmol). After 30 minutes of stirring, the reaction mixture was extracted with dichloromethane and washed with water. The organic phase was dried with anhydrous $MgSO_4$, concentrated, and the crude product was recrystallized from the chloroform and hexane mixture to give a 4*H*-dithieno[3,2-*b*:2',3'-*d*]pyrrole (0.41 g), with total reaction yield of 60%. **^{1}H NMR** (500 MHz, $CDCl_3$) δ 8.29 (s, 1H), 7.15 (d, J = 5.2 Hz, 2H), 7.03 (d, J = 5.3 Hz, 2H). **^{13}C NMR** (126 MHz, $CDCl_3$) δ 143.59, 123.40, 116.67, 112.20.

2.3. Synthesis of 4-(2-(2-(2-methoxyethoxy)ethoxy)ethyl)-4*H*-dithieno[3,2-*b*:2',3'-*d*]pyrrole (G-DTP)

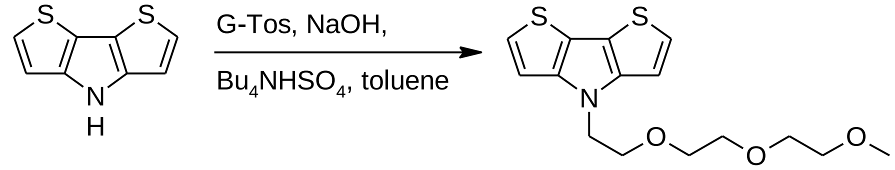

4*H*-dithieno[3,2-*b*:2',3'-*d*]pyrrole (H-DTP) (1.31 g, 7.30 mmol), anhydrous toluene (100 ml) and tetrabutylammonium hydrogen sulphate (VI) (0.79 g, 2.32 mmol) were added to a flask under argon. Next, a 50% aqueous solution of NaOH (16.69 g, 417.62 mmol) was added, stirred for 20 minutes and 2-(2-(2-methoxyethoxy)ethoxy)ethyl 4-methylbenzenesulfonate (G-Tos) (4.41 g, 13.91 mmol) was finally added to a reaction mixture. After 12 hours of stirring and heating to 90°C, the reaction mixture was cooled to room temperature, extracted with dichloromethane, washed with water, dried over anhydrous magnesium sulfate, and concentrated on a rotary evaporator. The crude product was purified by column flash chromatography using a dichloromethane and ethyl acetate solvent mixture (97:3 (*v/v*)). The pure product was a yellowish liquid (1.84 g, 78 % yield). **^{1}H NMR** (500 MHz, $CDCl_3$) δ 7.11 (d, *J* = 5.3 Hz, 2H), 7.05 (d, *J* = 5.3 Hz, 2H), 4.38 (t, *J* = 5.7 Hz, 2H), 3.84 (t, *J* = 5.7 Hz, 2H), 3.55 – 3.53 (m, 4H), 3.52 – 3.49 (m, 2H), 3.47 – 3.44 (m, 2H), 3.35 (s, 3H). **^{13}C NMR** (126 MHz, $CDCl_3$) δ 145.30, 122.84, 115.05, 111.46, 72.02, 71.02, 70.76, 70.67, 70.54, 59.14, 47.59.

2.4. Synthesis of 4-(2-(2-(2-methoxyethoxy)ethoxy)ethyl)-2-(tributylstannyl)-4*H*-dithieno[3,2-*b*:2',3'-*d*]pyrrole (G-DTP-$SnBu_3$)

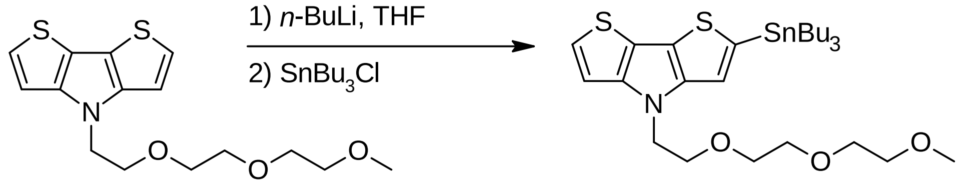

In a three-neck flask 4-(2-(2-(2-methoxyethoxy)ethoxy)ethyl)-4*H*-dithieno[3,2-*b*:2',3'-*d*]pyrrole (G-DTP) (1.0 g, 3.08 mmol) was dissolved in dry THF (15 ml). *n*-Butyllithium (1.23 ml, 3,08 mmol, 2.5 M in hexane) was added dropwise over 20 minutes at -78°C in the inert atmosphere of argon. The reaction was stirred for one hour at room temperature before adding tributyltin chloride (1.10 g, 3.38 mmol). The mixture was stirred over 12 hours at room temperature. The solution was extracted with dichloromethane and washed with water. The combined organic phases were dried over anhydrous $MgSO_4$, the drying agent drained, and the solvent evaporated. A 1.89 g (~80% purity) of brown oil was obtained, which was not subjected to further purification. **^{1}H NMR** (400 MHz, $CDCl_3$) δ 7.07 (d, *J* = 5.3 Hz, 1H), 7.04 (d, *J* = 5.3 Hz, 1H), 7.01 (s, 1H), 4.39 (t, *J* = 5.9 Hz, 2H), 3.85 (t, *J* = 5.9 Hz, 2H), 3.55 (d, *J* = 5.0 Hz, 4H), 3.53 – 3.49 (m, 2H), 3.48 – 3.44 (m, 2H), 3.35 (s, 3H), 1.63 – 1.56 (m, 6H), 1.35 (dt, *J* = 14.4, 7.3 Hz, 6H), 1.19 – 1.09 (m, 6H), 0.91 (t, *J* = 7.3 Hz, 9H). **^{13}C NMR** (101 MHz, $CDCl_3$) δ 147.99, 145.47, 135.52, 122.85, 122.43, 118.22, 115.02, 111.57, 72.01, 71.06, 70.76, 70.70, 70.52, 59.14, 47.51, 29.14, 27.43, 13.84, 11.05.

2.5. Synthesis of 2-(2-(2-(2-methoxyethoxy)ethoxy)ethyl)isoindoline-1,3-dione (G-Ftal)

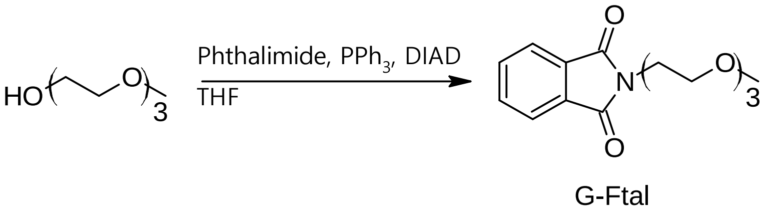

Phthalimide (14.12 g, 95.97 mmol), triethylene glycol monomethyl ether (13.08 g, 79.65 mmol), triphenylphosphine (25.17 g, 95.97 mmol), and THF (400 mL) were added to a three-neck flask purged with argon. The mixture was stirred for 15 minutes, and then diisopropyl azodicarboxylate (DIAD) (19.41 g, 95.97 mmol) was added. After 12 hours of stirring under an argon atmosphere, the ethanol (80 ml) was added into the reaction flask and stirred, and the solvents were then evaporated. The residue was dissolved in hexane and ethyl acetate mixture (1:1 (*v/v*)) and then stirred at 40°C for one hour. The resulting white precipitate was filtered, and the filtrate was concentrated and purified by column flash chromatography using a hexane and ethyl acetate mixture (1:1 (*v/v*)), yielding 18.77 g (80%) of colorless oil. ^{1}**H NMR** (500 MHz, $CDCl_3$) δ 7.82 (dd, *J* = 5.5, 3.0 Hz, 2H), 7.69 (dd, *J* = 5.5, 3.0 Hz, 2H), 3.88 (t, *J* = 5.8 Hz, 2H), 3.72 (t, *J* = 5.8 Hz, 2H), 3.65 – 3.62 (m, 2H), 3.60 – 3.55 (m, 4H), 3.47 – 3.44 (m, 2H), 3.32 (s, 3H). ^{13}**C NMR** (126 MHz, $CDCl_3$) δ 168.33, 134.00, 132.25, 123.31, 71.97, 70.67, 70.64, 70.21, 68.00, 59.09, 37.36.

2.6. Synthesis of 2-(2-(2-methoxyethoxy)ethoxy)ethan-1-amine (G-NH$_2$)

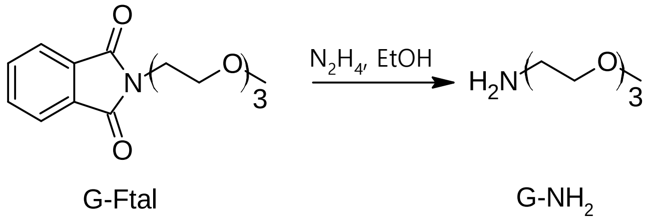

2-(2-(2-(2-methoxyethoxy)ethoxy)ethyl)isoindoline-1,3-dione (G-Ftal) (18.74 g, 63.89 mmol) and ethanol (220 mL) were added into a three-neck flask. Hydrazine monohydrate (3.84 g, 76.68 mmol) was then added dropwise and stirred at 80°C for 18 hours until the solution turned yellow, and a white precipitate formed. The reaction mixture was refluxed for an additional 5 hours. After cooling to room temperature, concentrated HCl (19 ml) was added to the reaction mixture, and the reflux continued for one hour. The precipitate was then filtered, and the filtrate was concentrated. The residue was dissolved in 150 ml of water, and the pH was adjusted to ≈ 11 with the addition of a 1 M NaOH solution. The mixture was extracted with brine and dichloromethane (5 x 200 mL). The crude product was purified by distillation under reduced pressure (t = 110°C, p = $1.3 \cdot 10^{-3}$ mbar, Kugelrohr - BUCHI), yielding 51% (5.32 g) of colorless liquid. ^{1}**H NMR** (400 MHz, $CDCl_3$) δ 3.66 – 3.60 (m, 6H), 3.55 – 3.52 (m, 2H), 3.51 – 3.48 (m, 2H), 3.36 (s, 3H), 2.87 – 2.83 (m, 2H). ^{13}**C NMR** (101 MHz, $CDCl_3$) δ 73.51, 72.05, 70.72, 70.65, 70.38, 59.16, 41.89.

2.7. Synthesis of 4,9-dibromo-2,7-dibutylbenzo[*lmn*][3,8]phenanthroline-1,3,6,8(2*H*,7*H*)-tetraone (Bu-NDI)

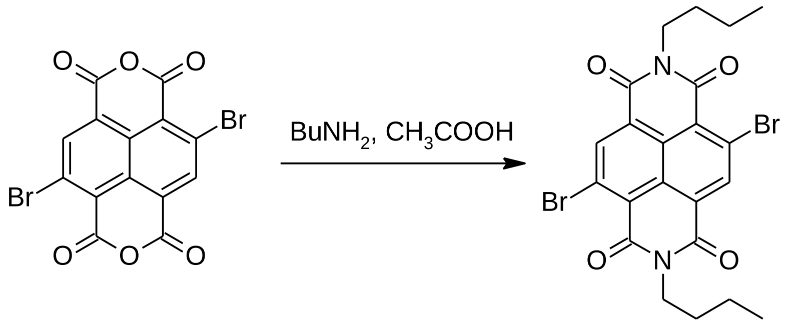

2,6-Dibromo-1,4,5,8-naphthalenetetracarboxylic acid dianhydride (3.00 g, 7.03 mmol), glacial acetic acid (100 ml) and *n*-butylamine (2.06 mg, 28.13 mmol) were added to argon-filled three-neck flask. The mixture was heated to 100°C for 12 hours. After this time, the precipitate was filtered and dried in a vacuum oven to yield 3.41 g (90%) of yellow solid. ^{1}**H NMR** (500 MHz, $CDCl_3$) δ 8.99 (s, 2H), 4.22 – 4.18 (m, 4H), 1.76 –1.69 (m, 4H), 1.50- 1.42 (m, 4H), 0.99 (t, J = 7.4 Hz, 6H). ^{13}**C NMR** (126 MHz, $CDCl_3$) δ 160.91, 160.89, 139.22, 128.48, 127.87, 125.50, 124.23, 41.53, 30.09, 20.50, 13.92.

2.8. Synthesis of 4,9-dibromo-2,7-bis(2-(2-(2-methoxyethoxy)ethoxy)ethyl) benzo[*lmn*][3,8] phenan-throline-1,3,6,8(2*H*,7*H*)-tetraone (G-NDI)

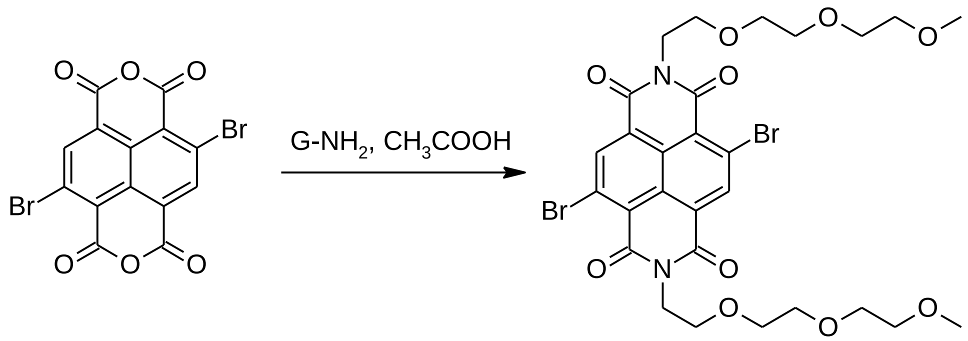

In a three-neck flask, 2,6-dibromo-1,4,5,8-naphthalenetetracarboxylic acid dianhydride (4.00 g, 9.38 mmol), glacial acetic acid (150 ml), and 2-(2-(2-methoxyethoxy)ethoxy)ethan-1-amine ($G\text{-}NH_2$) (6.12 g, 37.51 mmol) were added, and the mixture was heated to 90°C for 16 hours under argon atmosphere. After this time, the reaction mixture was poured into a beaker with 2 liters of water. The resulting precipitate was filtered and washed with water. The crude product was purified using a chromatographic column using a dichloromethane and acetone solvent mixture (3:1 (*v/v*)) and yielding 5.71 g of a yellow solid (85% yield). ^{1}**H NMR** (500 MHz, $CDCl_3$) δ 8.98 (s, 2H), 4.46 (t, J = 5.8 Hz, 4H), 3.85 (t, J = 5.8 Hz, 4H), 3.70 (dd, J = 5.7, 3.8 Hz, 4H), 3.62 (dd, J = 5.6, 3.8 Hz, 4H), 3.59 – 3.57 (m, 4H), 3.48 – 3.46 (m, 4H), 3.32 (s, 6H). ^{13}**C NMR** (126 MHz, $CDCl_3$) δ 161.00, 160.90, 139.19, 128.50, 127.93, 125.48, 124.26, 72.03, 70.77, 70.68, 70.25, 67.73, 59.17, 40.17.

2.9. Synthesis of 2,7-dibutyl-4,9-bis(4-(2-(2-(2-methoxyethoxy)ethoxy)ethyl)-4*H*-dithieno[3,2-b:2',3'-d]pyrrol-2-yl)benzo[*lmn*][3,8]phenanthroline-1,3,6,8(*2H*,7*H*)-tetraone *(G-DTP-Bu-NDI)*

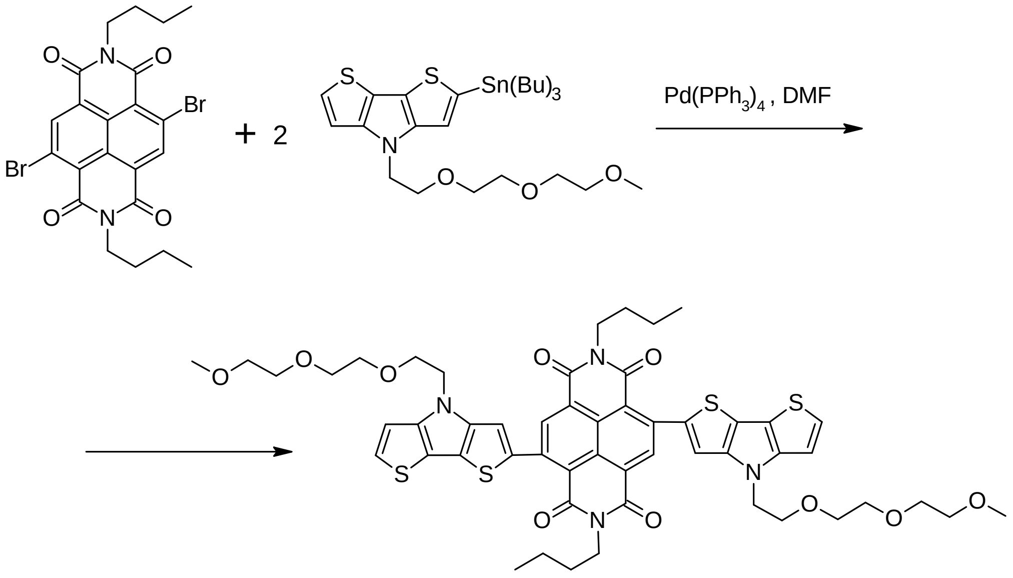

4,9-dibromo-2,7-dibutylbenzo[*lmn*][3,8]phenanthroline-1,3,6,8(2*H*,7*H*)-tetraone (Bu-NDI) (0.28 g, 0.52 mmol) and DMF (40 ml) were added to a flask under argon atmosphere and the mixture was heated to 60°C during stirring until the substrate dissolved. Then tetrakis(triphenylphosphine)palladium(0) (0.06 g, 0.05 mmol) and 4-(2-(2-(2-methoxyethoxy)ethoxy)ethyl)-2-(tributylstannyl)-4*H*-dithieno[3,2-*b*:2',3'-*d*]pyrrole (G-DTP-SnBu$_3$) (0.80 g, 1.30 mmol) were added and a mixture was heated at 100°C for 24 h. Upon cooling to room temperature, the reaction mixture was diluted with dichloromethane (100 ml), and the resulting mixture was washed with water (50 ml × 2), dried over anhydrous magnesium sulfate, and concentrated on a rotary evaporator. The crude product was passed through silica pad using a dichloromethane and acetonitrile mixture (5:1 (*v/v*)) as eluent. The product was then purified by column flash chromatography using a dichloromethane and ethyl acetate (4:1 (*v/v*)) mixture as eluent. The final product was a blue solid (0.14 g), obtained with a yield of 27%. **^{1}H NMR** (400 MHz, CDCl$_3$) δ 8.85 (s, 2H), 7.40 (s, 2H), 7.21 (d, *J* = 5.4 Hz, 2H), 7.10 – 7.05 (m, 2H), 4.42 (t, *J* = 5.6 Hz, 4H), 4.18 – 4.11 (m, 4H), 3.89 (t, *J* = 5.6 Hz, 4H), 3.62 – 3.57 (m, 8H), 3.55 – 3.52 (m, 4H), 3.47 – 3.44 (m, 4H), 3.33 (s, 6H), 1.74 – 1.65 (m, 4H), 1.48 – 1.36 (m, 4H), 0.95 (t, *J* = 7.4 Hz, 6H). **^{13}C NMR** (101 MHz, CDCl$_3$) δ 162.52, 162.40, 146.24, 145.01, 140.73, 137.75, 136.96, 127.62, 125.07, 124.86, 122.25, 118.57, 115.51, 113.36, 111.52, 72.01, 71.05, 70.79, 70.68, 70.55, 59.14, 47.67, 41.10, 30.25, 20.50, 14.00. **Elemental analysis**: Calcd. for $C_{52}H_{56}N_4O_{10}S_4$: C 60.92; H 5.51; N 5.46; S 12.51. Found: C 60.83; H 5.42; N 5.39; S 12.40. **HRMS (ESI)**: calcd. for $C_{52}H_{56}N_4O_{10}S_8$: 1047.2777; found: 1047.2760.

2.10. Synthesis of 2,7-bis(2-(2-(2-methoxyethoxy)ethoxy)ethyl)-4,9-bis(4-(2-(2-(2-methoxyethoxy) ethoxy)ethyl)-4*H*-dithieno[3,2-b:2',3'-d]pyrrol-2-yl)benzo[*lmn*][3,8]phenanthroline-1,3,6,8(2*H*, 7*H*)-tetraone (G-DTP-G-NDI)

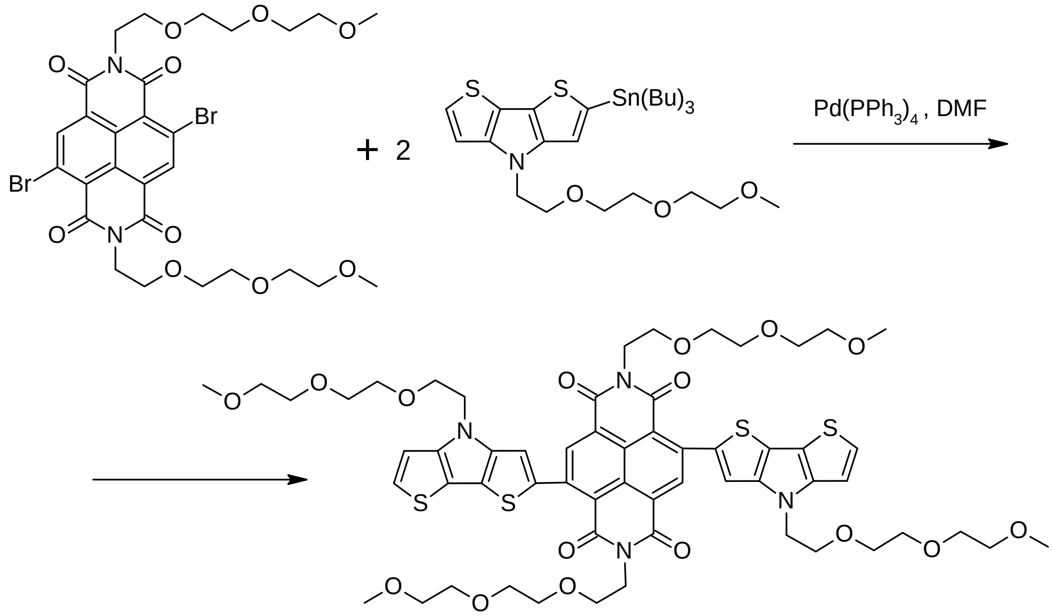

4,9-dibromo-2,7-bis(2-(2-(2-methoxyethoxy)ethoxy)ethyl)benzo[*lmn*][3,8]phenanthroline-1,3,6,8(2*H*, 7*H*)-tetraone (G-NDI) (0.77 g, 1.07 mmol), tetrakis(triphenylphosphine)palladium(0) (0.12 g, 0.11 mmol), 4-(2-(2-(2-methoxyethoxy)ethoxy)ethyl)-2-(tributylstannyl)-4*H*-dithieno[3,2-*b*:2',3'-*d*]pyrrole (G-DTP-$SnBu_3$) (1.51 g, 2.46 mmol) and anhydrous toluene (50 ml) were added to a flask under argon atmosphere. The mixture was stirred and heated at 110°C for 12 h. Upon cooling to room temperature, the reaction mixture was diluted with dichloromethane (100 ml), and the resulting mixture was washed with water (50 ml × 3), dried over anhydrous magnesium sulfate, and concentrated on a rotary evaporator. The crude product was purified by column flash chromatography using a dichloromethane and acetone solvent mixture (3:1 (*v/v*)). The pure product was obtained as a blue solid (0.77 g, 60% yield). **^{1}H NMR** (400 MHz, $CDCl_3$) δ 8.85 (s, 2H), 7.42 (s, 2H), 7.21 (d, *J* = 5.3 Hz, 2H), 7.09 (d, *J* = 5.3 Hz, 2H), 4.45 – 4.38 (m, 8H), 3.90 (t, *J* = 5.6 Hz, 4H), 3.80 (t, *J* = 5.9 Hz, 4H), 3.70 – 3.66 (m, 4H), 3.63 – 3.56 (m, 16H), 3.55 – 3.52 (m, 4H), 3.47 – 3.44 (m, 8H), 3.33 (s, 6H), 3.30 (s, 6H). **^{13}C NMR** (101 MHz, $CDCl_3$) δ 162.58, 162.44, 146.34, 145.04, 140.78, 137.58, 136.97, 127.69, 125.06, 124.89, 122.17, 118.63, 115.49, 113.49, 111.61, 72.02, 71.05, 70.79, 70.75, 70.67, 70.56, 70.28, 67.98, 59.13, 47.69, 39.87. **Elemental analysis**: Calcd. for $C_{52}H_{56}N_4O_{10}S_4$: C 57.79; H 5.69; N 4.65; S 10.64. Found: C 57.70; H 5.66; N 4.80; S 10.71. **HRMS (ESI)**: calcd. for $C_{58}H_{68}N_4O_{16}S_4$: 1227.3411; found: 1227.3398.

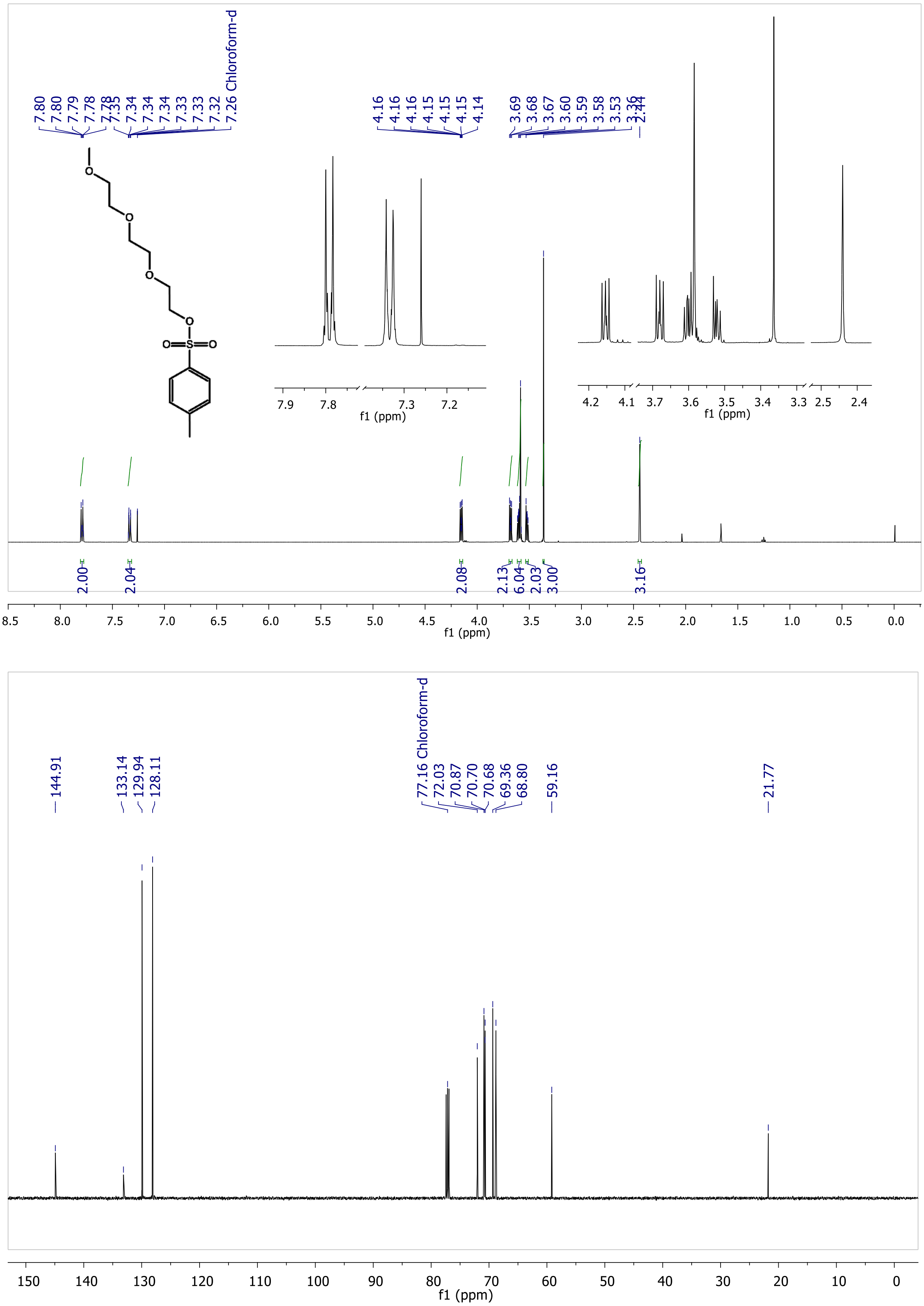

Figure S1. ^{1}H NMR and ^{13}C NMR spectra ($CDCl_3$) of **G-Tos**.

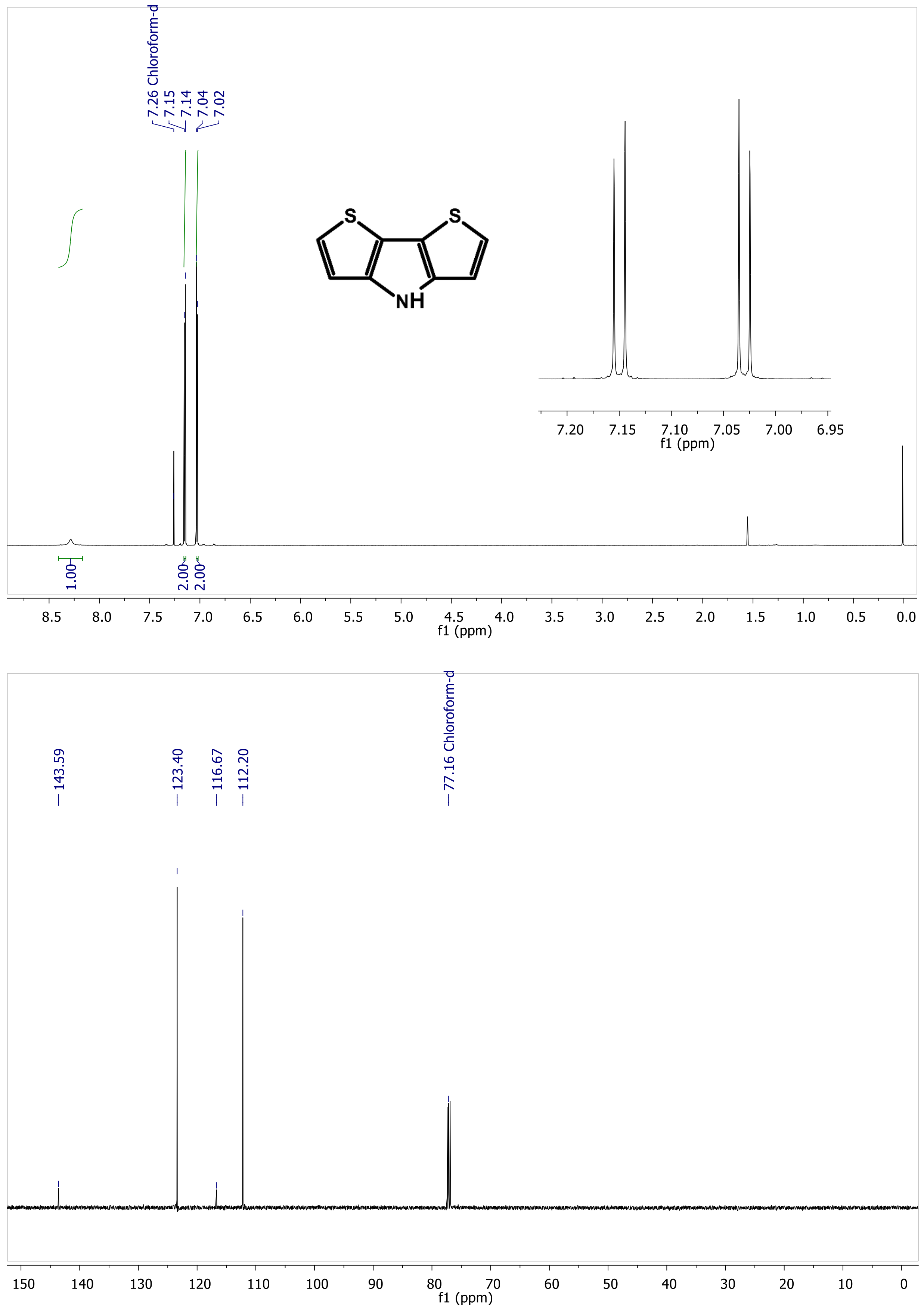

Figure S2. ^{1}H NMR and ^{13}C NMR spectra ($CDCl_3$) of **H-DTP**.

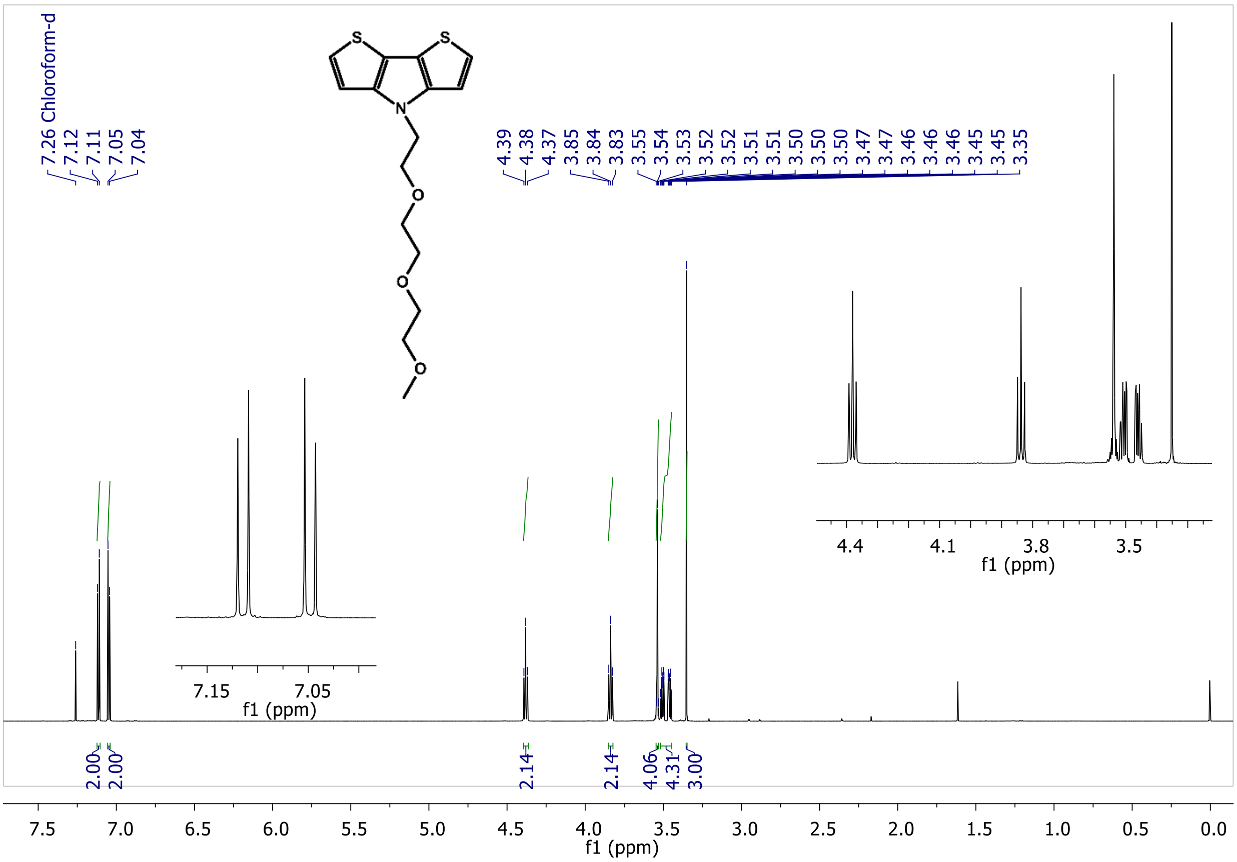

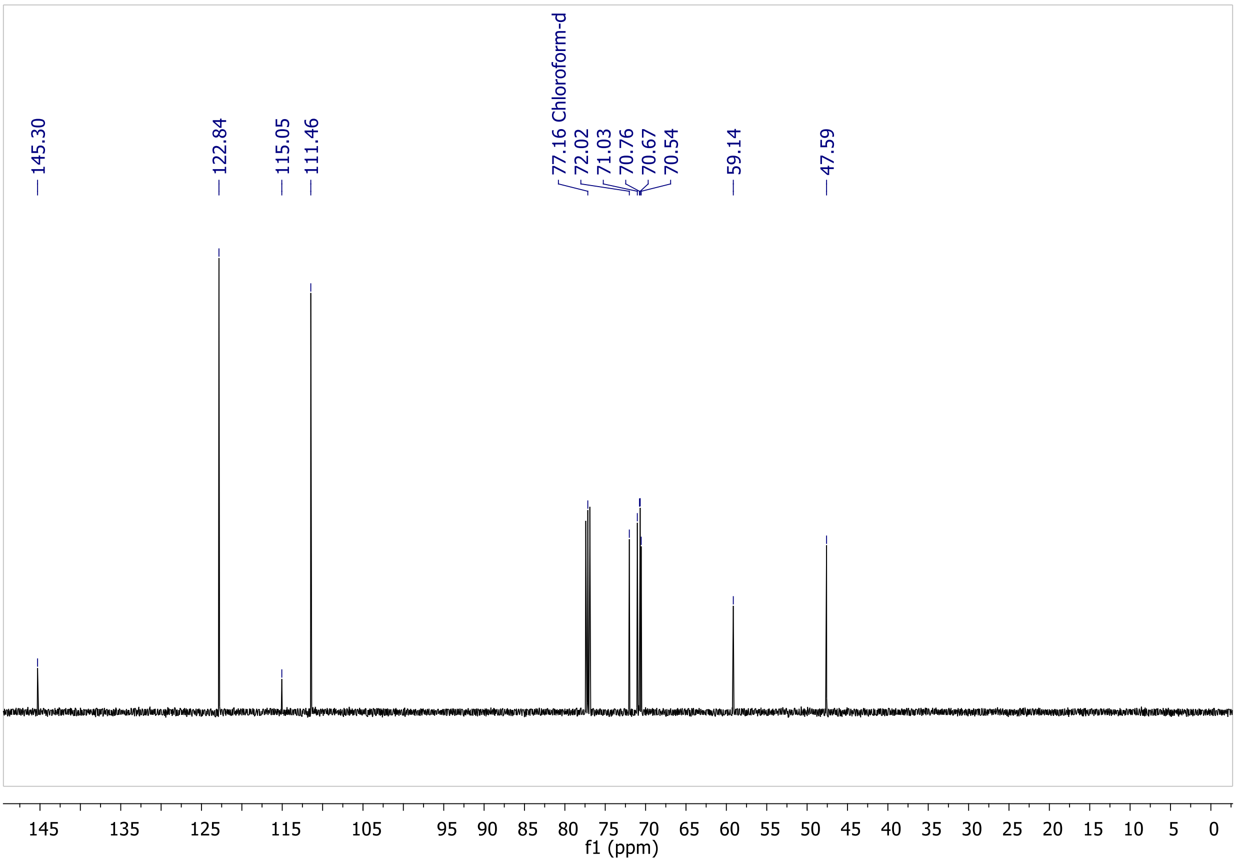

Figure S3. ^{1}H NMR and ^{13}C NMR spectra ($CDCl_3$) of **G-DTP**.

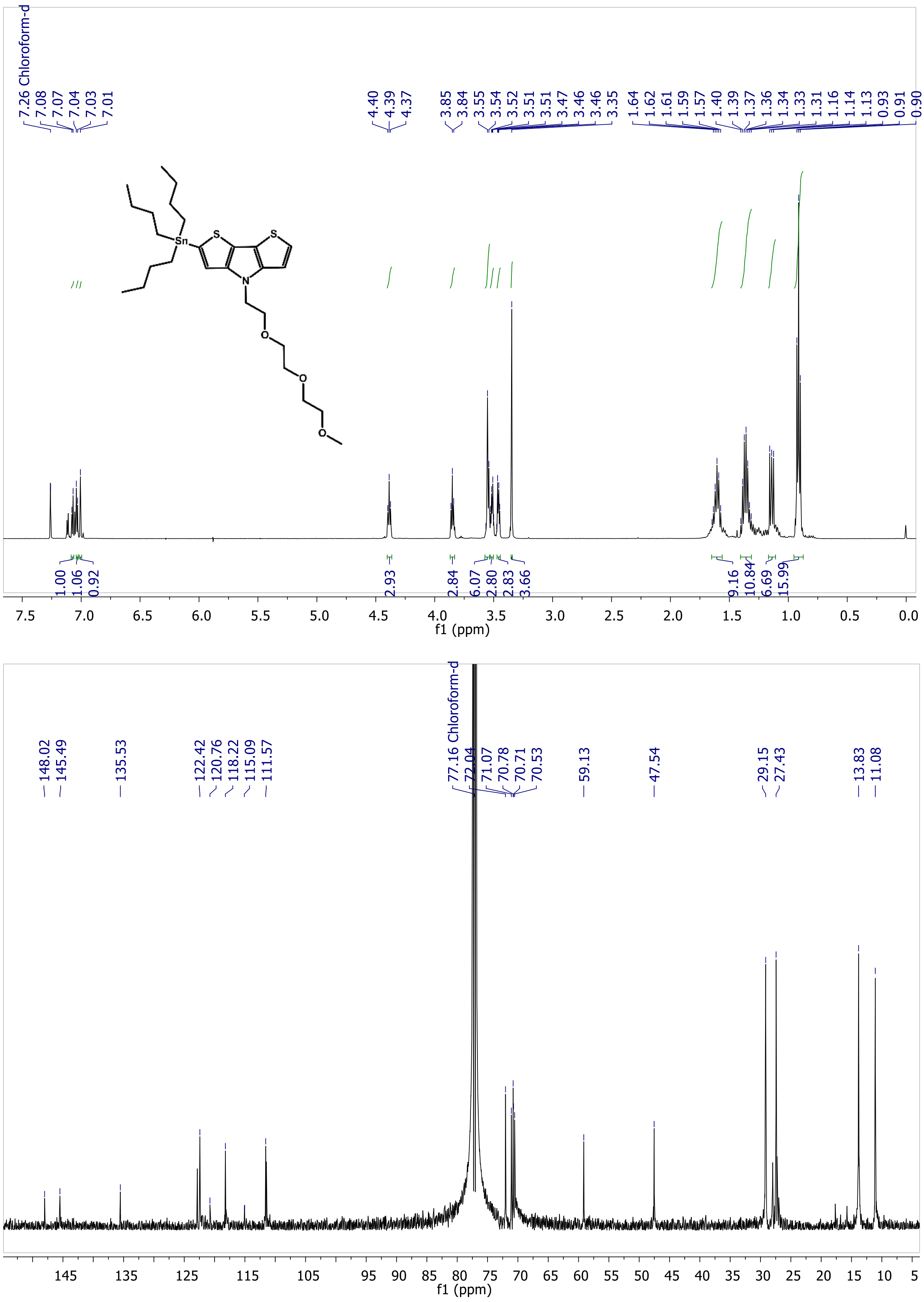

Figure S4. ^{1}H NMR and ^{13}C NMR spectra ($CDCl_3$) of **G-DTP-SnBu$_3$**.

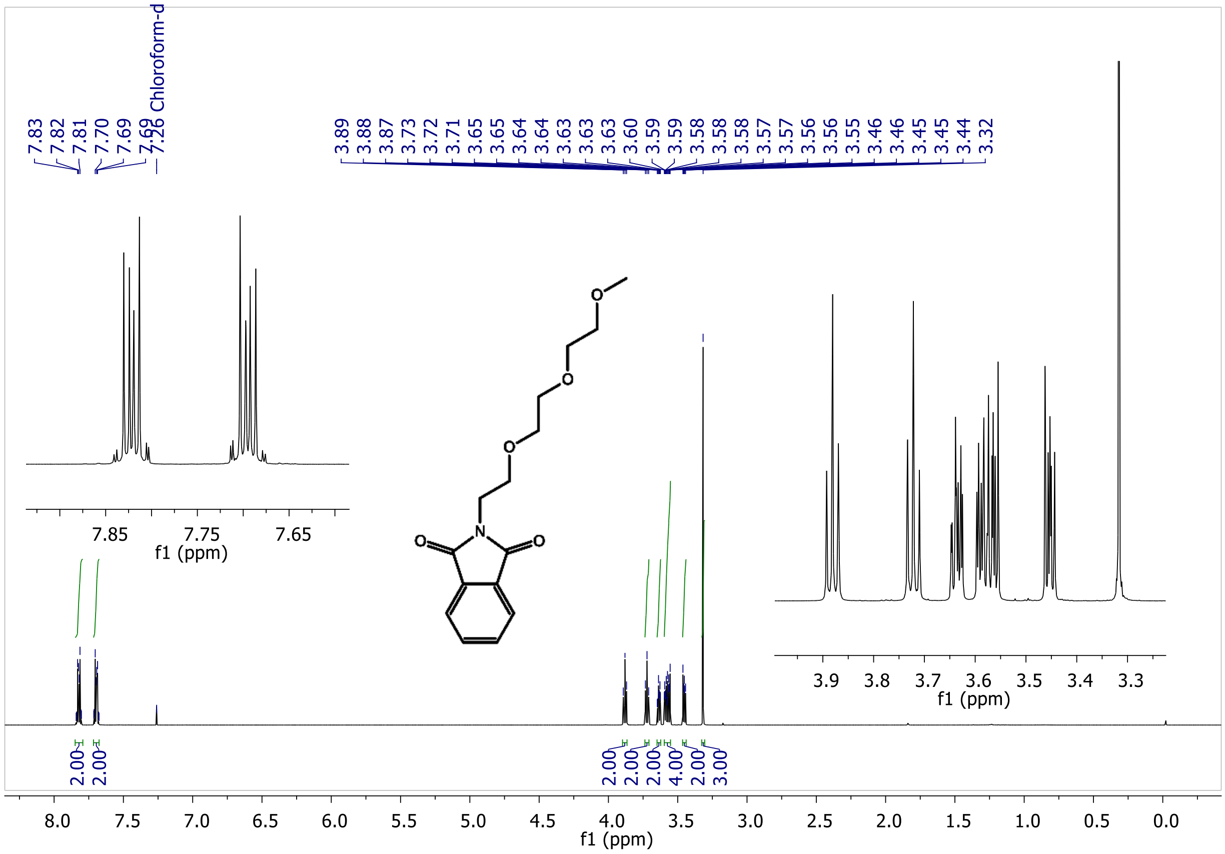

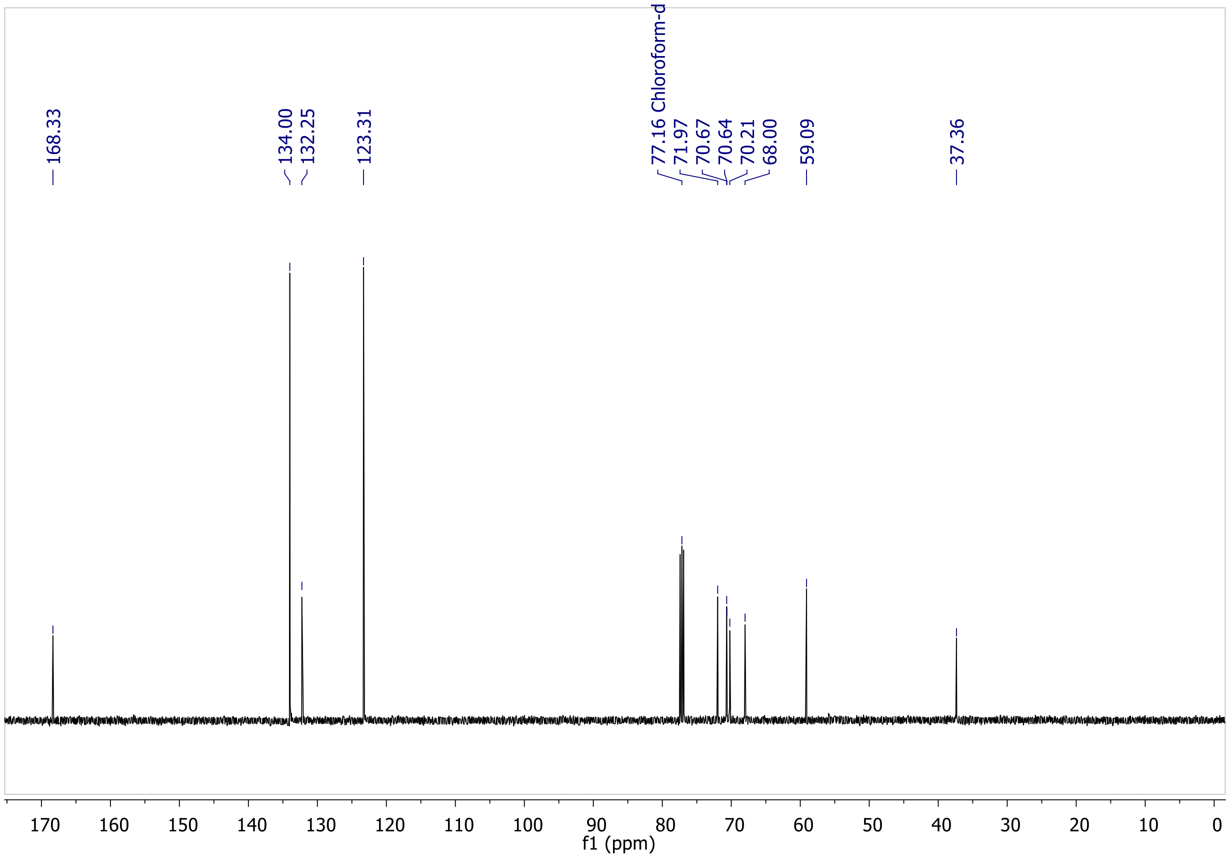

Figure S5. ^{1}H NMR and ^{13}C NMR spectra ($CDCl_3$) of **G-Ftal**.

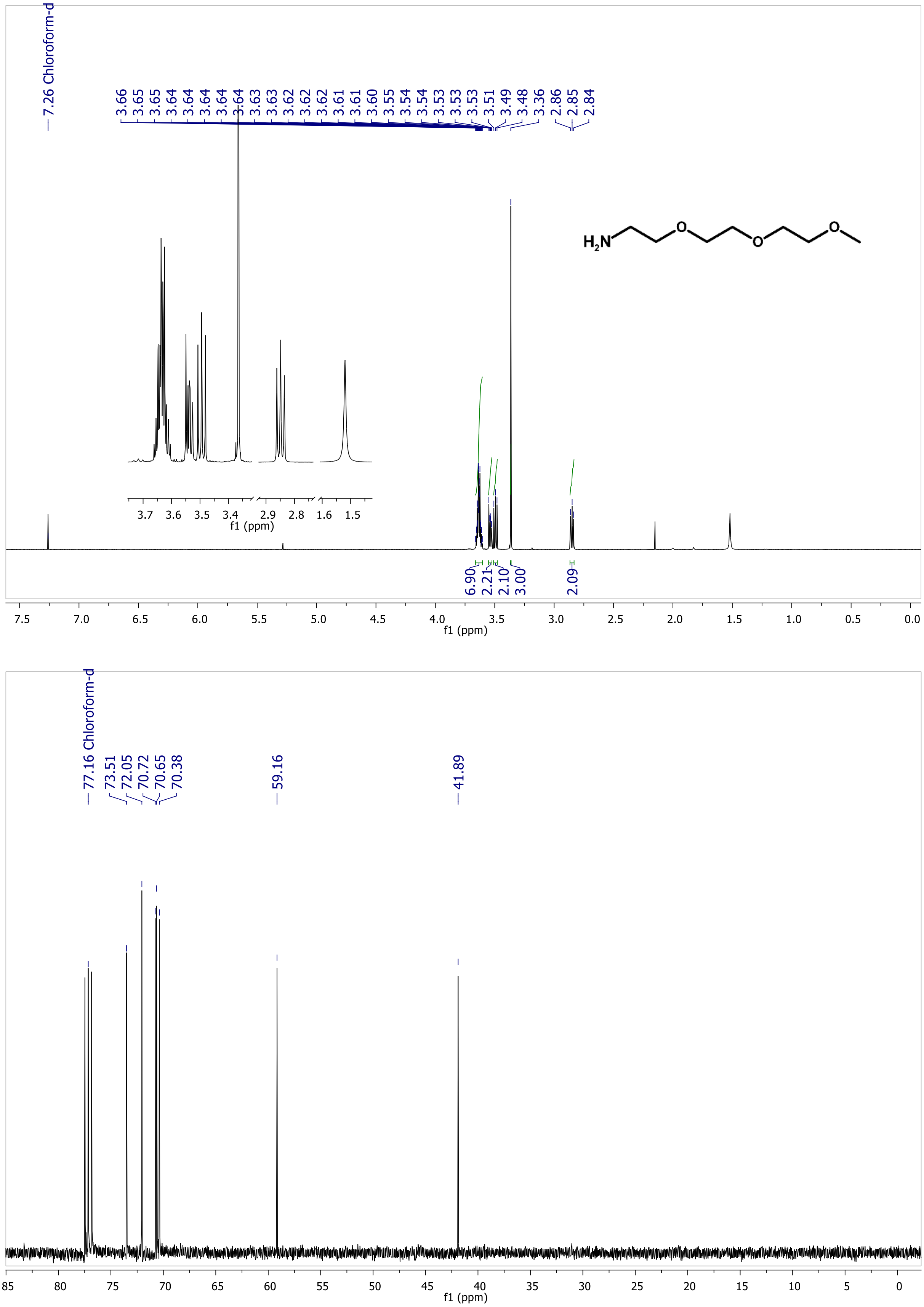

Figure S6. ^{1}H NMR and ^{13}C NMR spectra ($CDCl_3$) of **G-NH$_2$**.

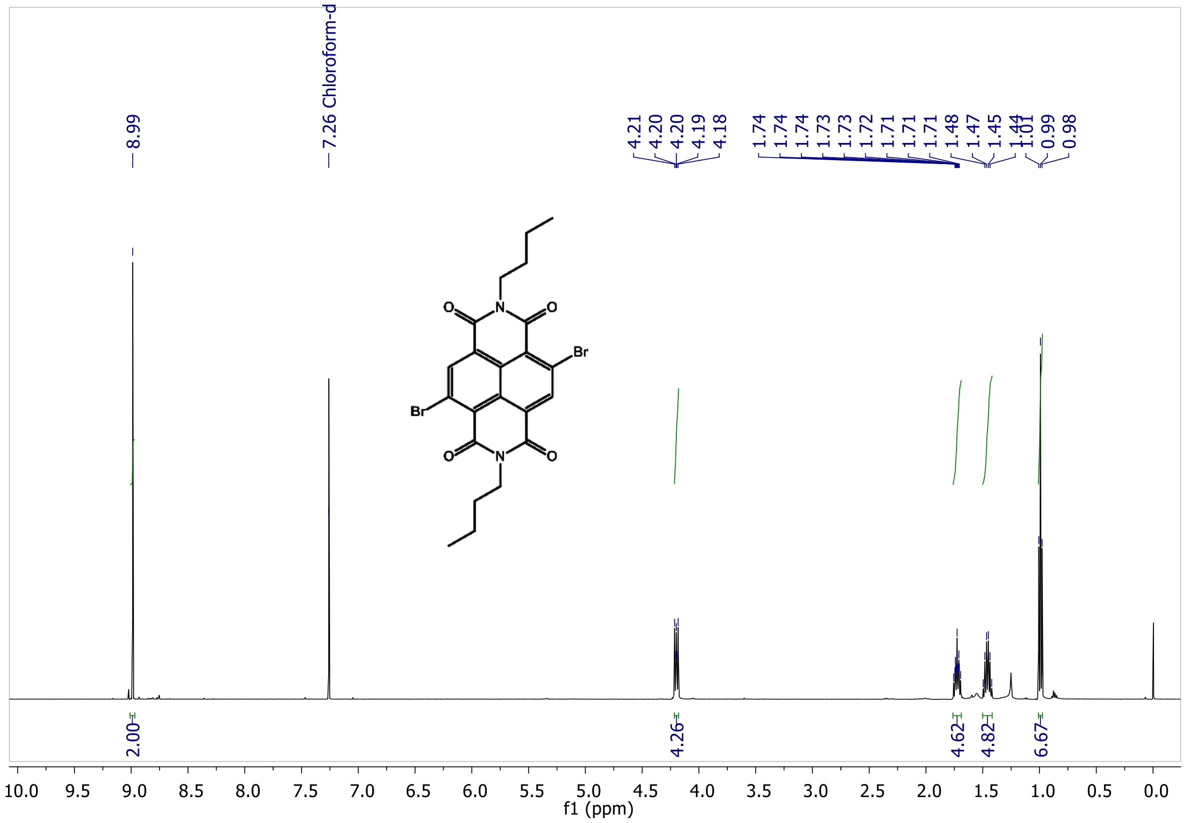

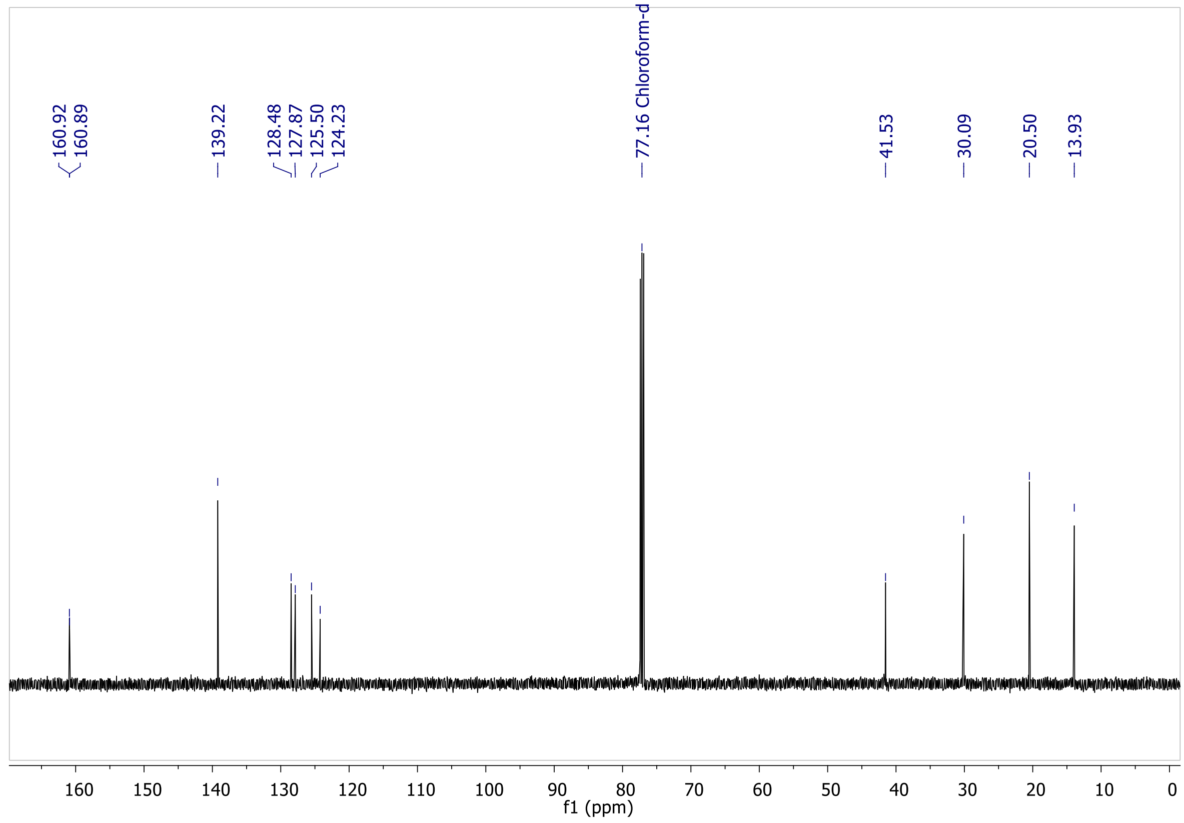

Figure S7. ^{1}H NMR and ^{13}C NMR spectra ($CDCl_3$) of **Bu-NDI**.

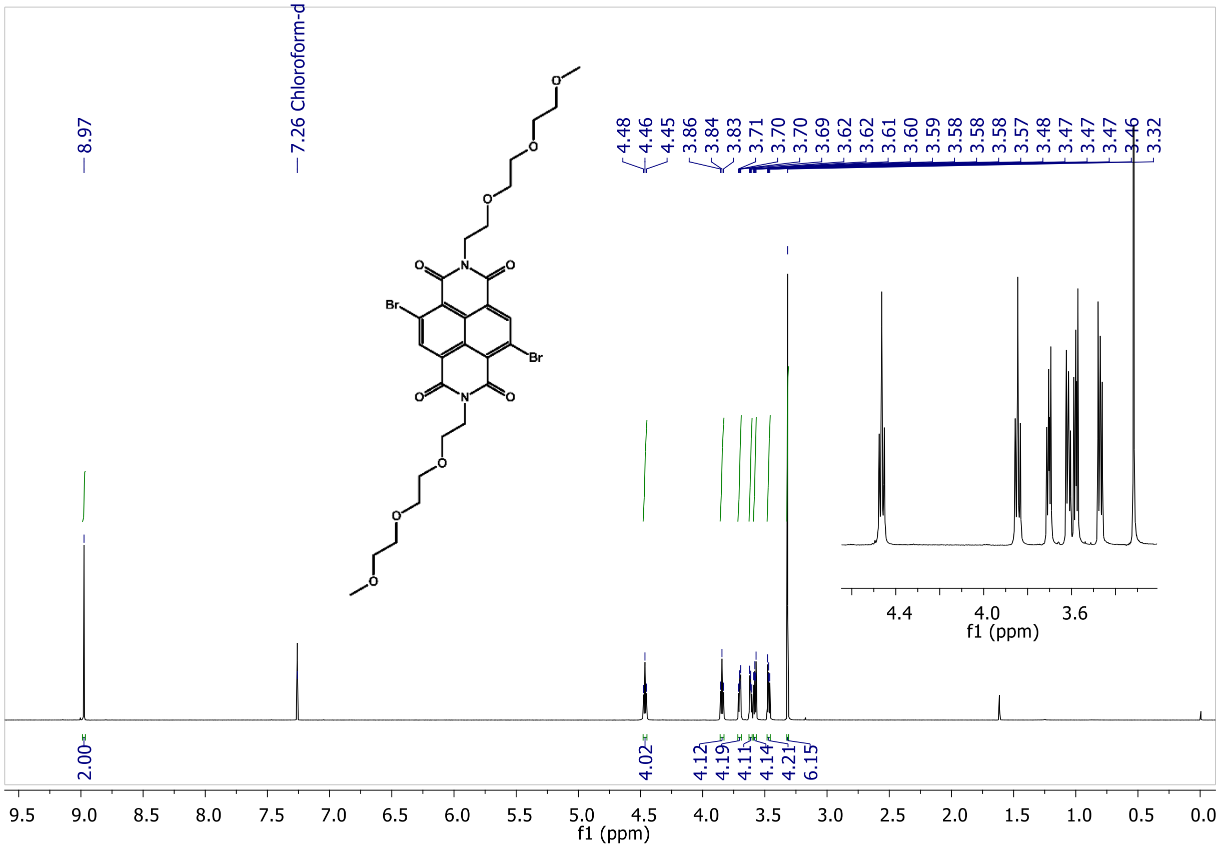

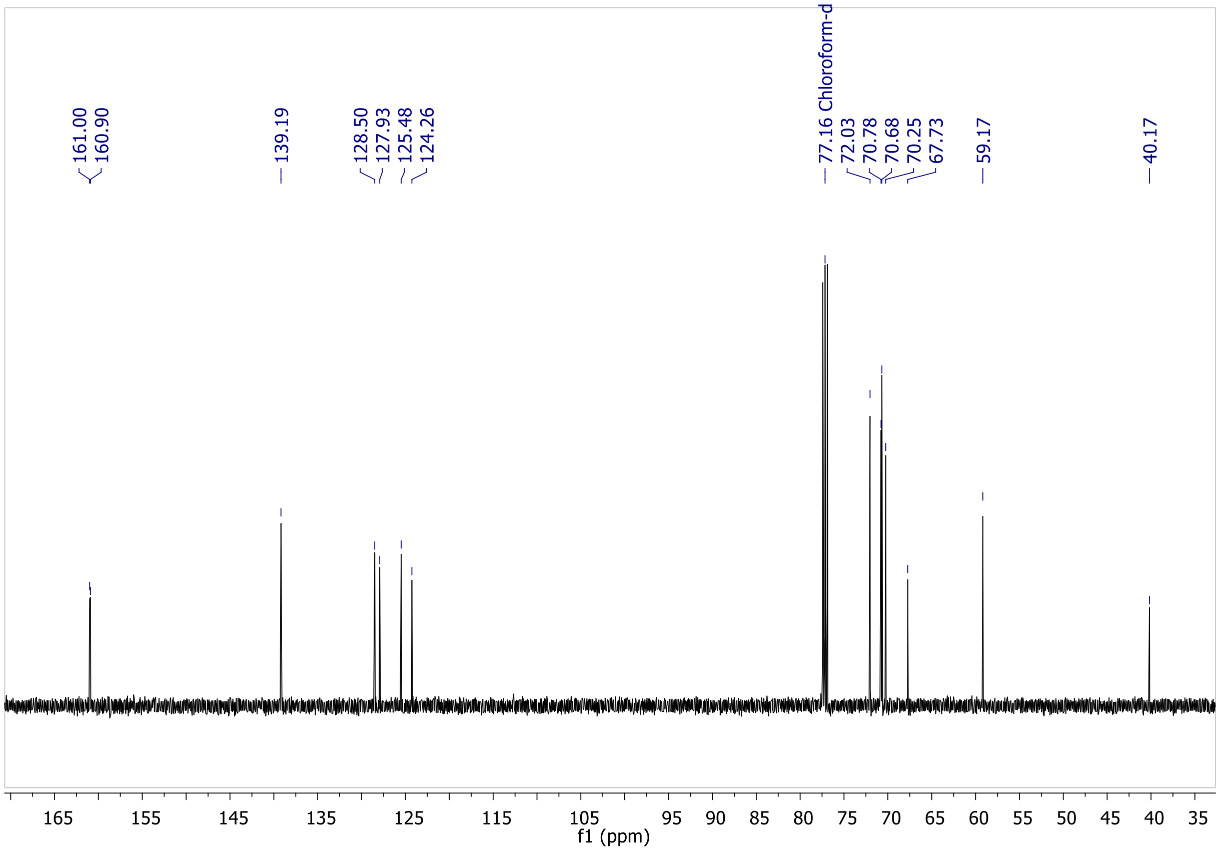

Figure S8. ^{1}H NMR and ^{13}C NMR spectra ($CDCl_3$) of **G-NDI**.

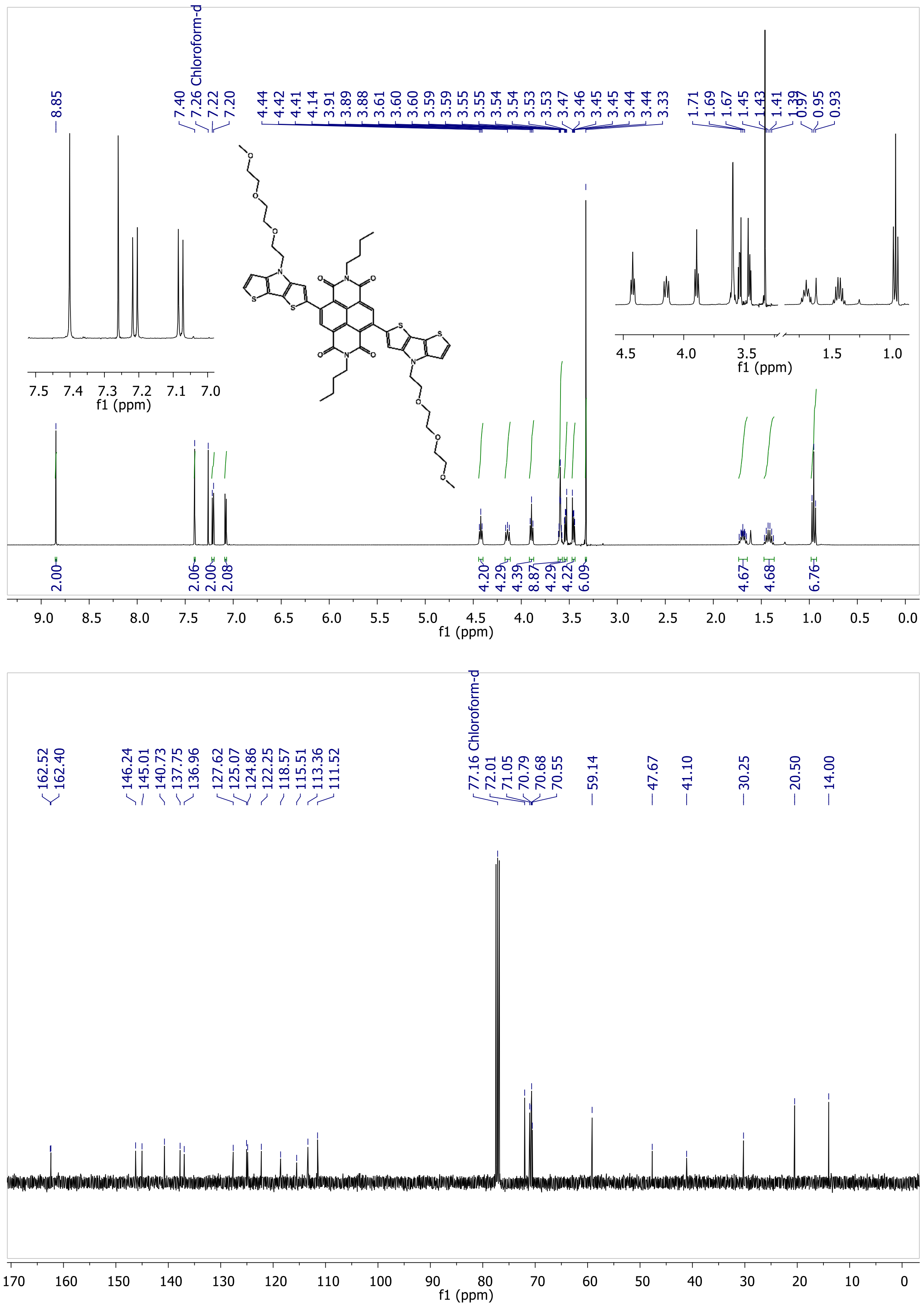

Figure S9. ^{1}H NMR and ^{13}C NMR spectra ($CDCl_3$) of **G-DTP-Bu-NDI**.

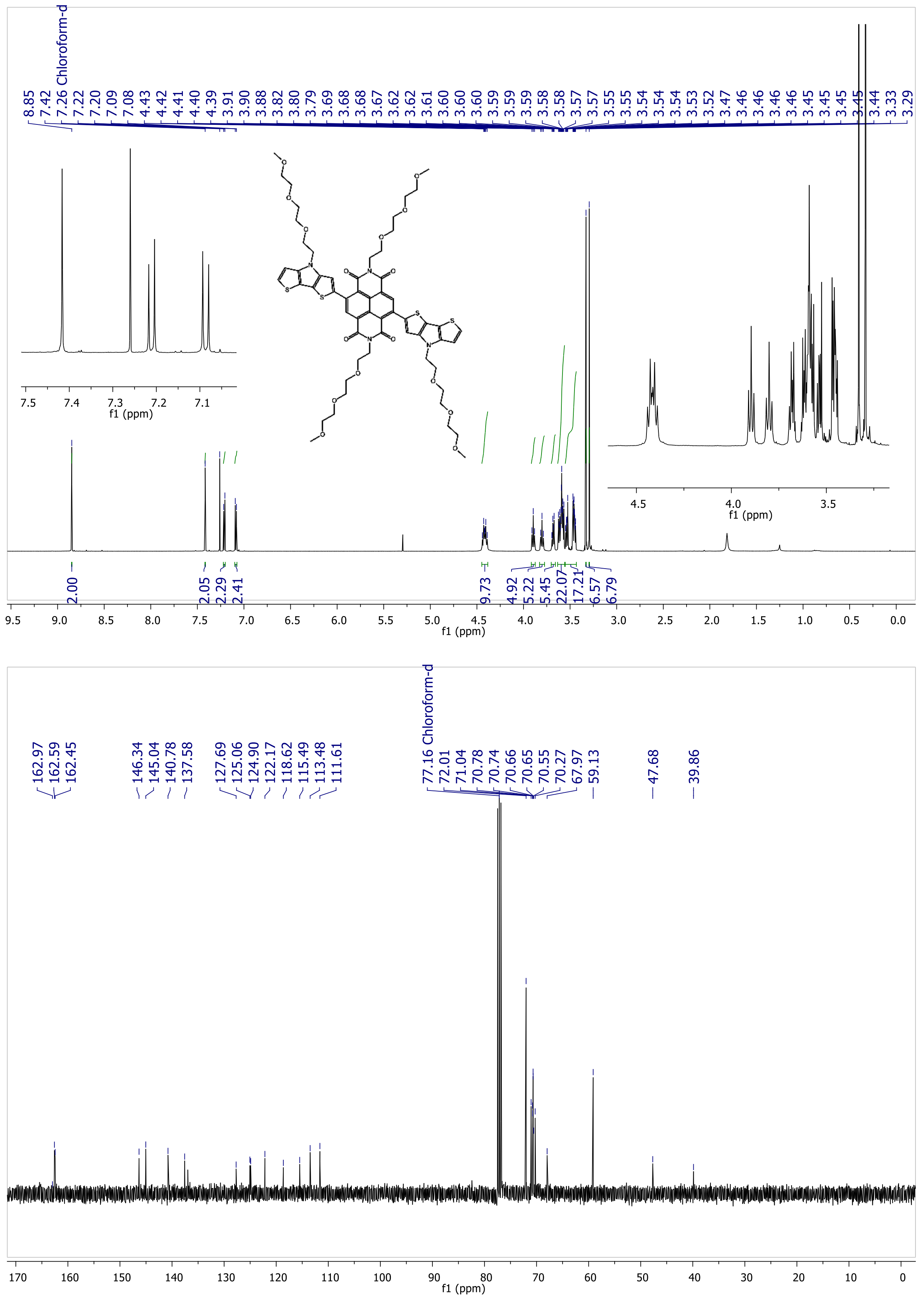

Figure S10. ^{1}H NMR and ^{13}C NMR spectra ($CDCl_3$) of **G-DTP-G-NDI**.

3. **Spectroscopic, electrochemical and spectroelectrochemical studies**

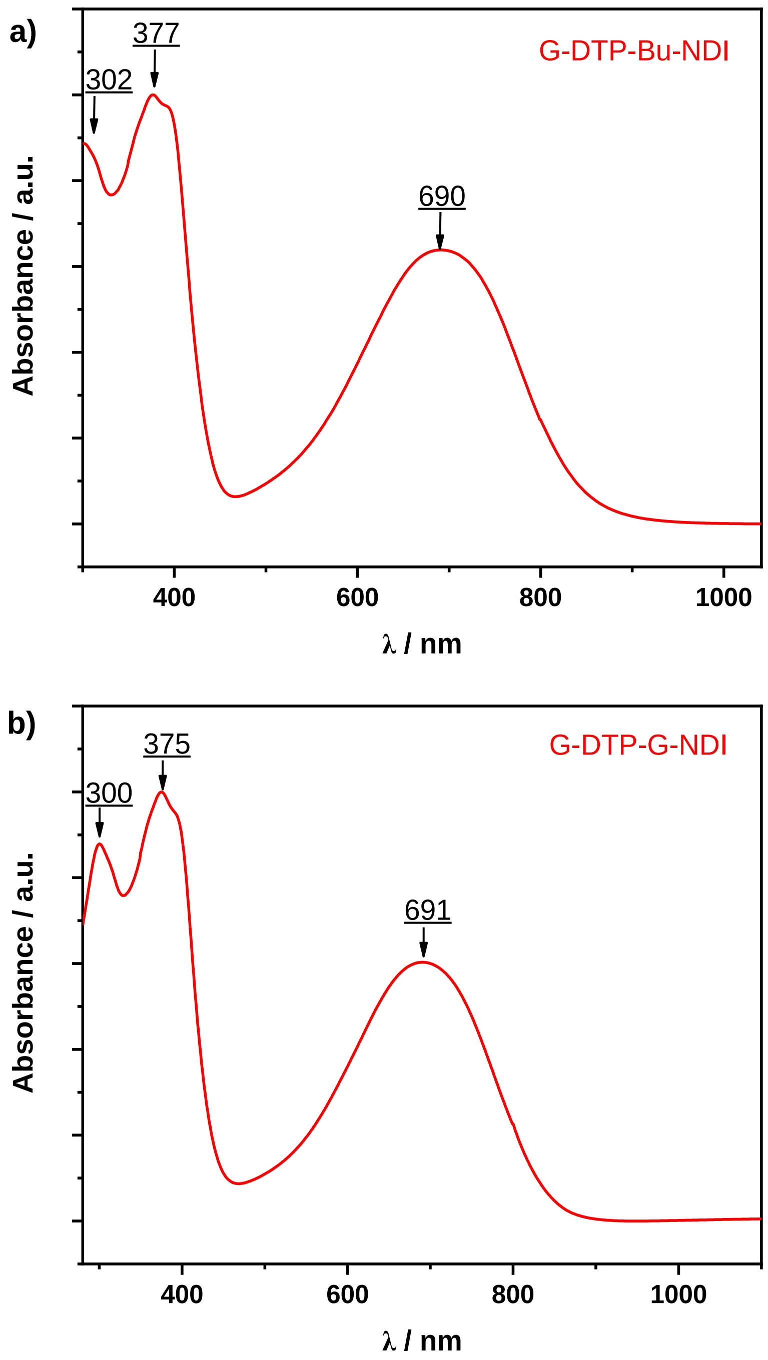

Figure S11. UV-vis spectra of drop-casted thin films of **G-DTP-Bu-NDI** (a) and **G-DTP-G-NDI** (b).

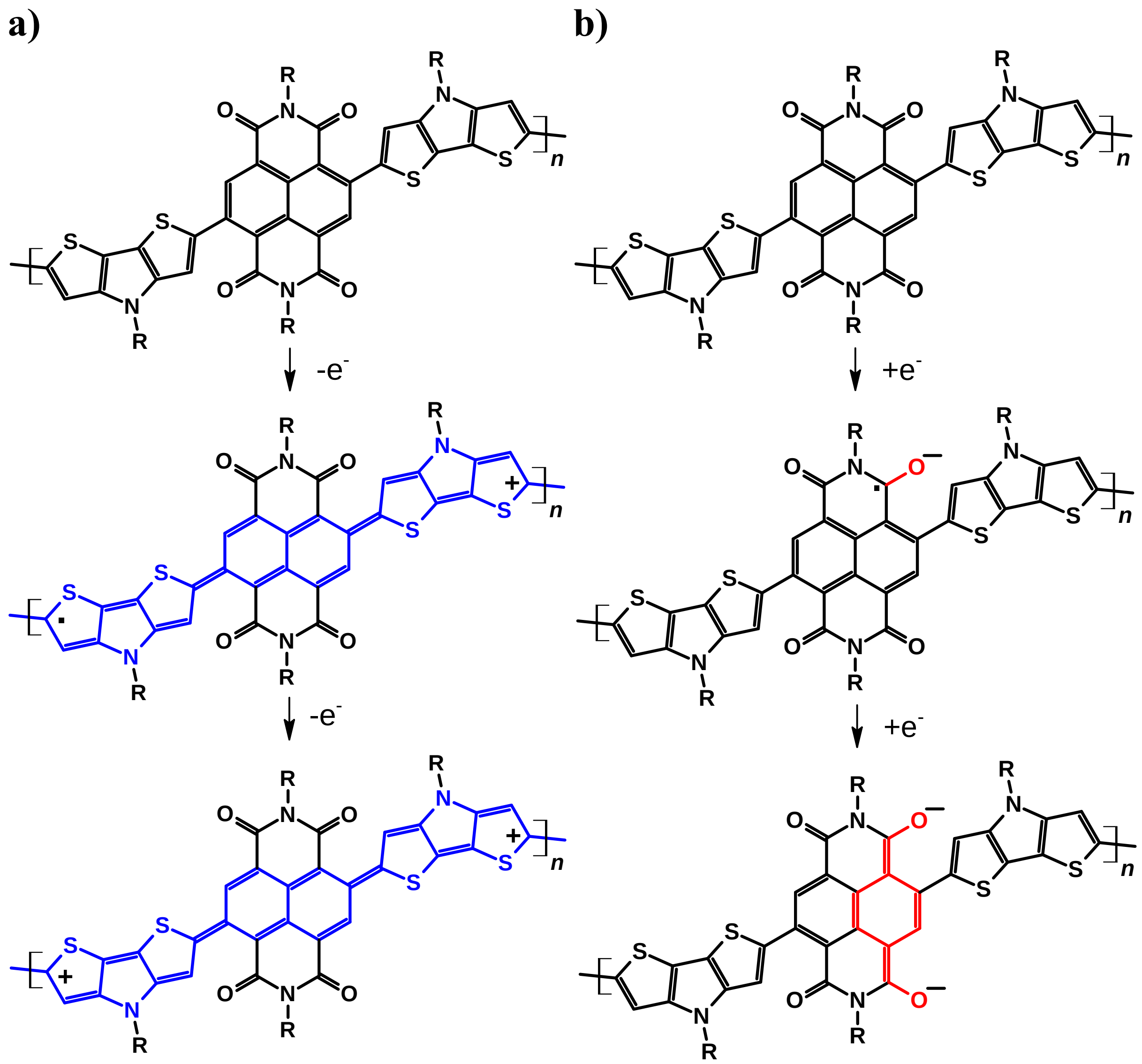

Figure S12. Scheme of p- and n-doping of **poly(G-DTP-(Bu/G)-NDI)**: **(a)** Two-electron oxidation resulting in the formation of a radical cation in the first step followed by a spinless dication in the second step (blue parts of the structure denote global changes in electron density distribution); **(b)** two-electron reduction leading to the formation of a radical anion in the first step followed by a spinless dianion in the second step (red parts of the structure denote local changes in electron density distribution).

Table S1. Cyclic voltammetry data, ionization potentials (IP), electron affinities (EA) and band gaps (E_g^{el}) of studied compounds. Potentials given vs Fc/Fc$^+$.

	Negative potentials range [V]					Positive potentials range [V]				IP	EA	E_g^{el}
**Compound**	E_{red1}	E_{red2}	E_{ox2}	E_{ox1}	E_{red}^{onset}	E_{ox1}	E_{red1}	E_{red2}	E_{ox}^{onset}		[eV]	
**G-DTP-Bu-NDI**	-1.11	-1.53	-1.41	-1.02	-1.00	0.56	0.42	0.12	0.39	5.24	-3.65	1.59
**G-DTP-G-NDI**	-1.09	-1.51	-1.33	-0.98	-0.98	0.51	0.39	0.10	0.37	5.36	-3.68	1.68

Data calculated: (a) from the onset of the first oxidation peak, using equation: $IP = |e|1{,}15E_{ox}(onset) + 4{,}79$; (b) from the onset of the first reduction peak using equation: $EA = -(|e|1{,}18E_{red}(onset) + 4{,}83)$; (c) in accordance with the equation $E_g^{el} = |E_{IP} + E_{EA}|$.

Table S2. Cyclic voltammetry data, ionization potentials (IP), electron affinities (EA) and band gaps (E_g^{el}) of the studied electropolymerization products. Potentials given vs. Fc/Fc^+

	Negative potentials range [V]					Positive potentials range [V]					IP	EA	E_g^{el}
**Polymer**	E_{red1}	E_{red2}	E_{ox2}	E_{ox1}	E_{red}^{onset}	E_{ox1}	E_{ox2}	E_{red2}	E_{red1}	E_{ox}^{onset}		[eV]	
**poly(G-DTP-Bu-NDI)**	-1.16	-1.53	-1.33	-0.98	-0.96	0.19	0.60	0.40	0.06	-0.18	4.58	-3.70	0.88
**poly(G-DTP-G-NDI)**	-1.04	-1.40	-1.32	-0.98	-0.92	0.16	0.58	0.50	0.12	-0.15	4.61	-3.74	0.87

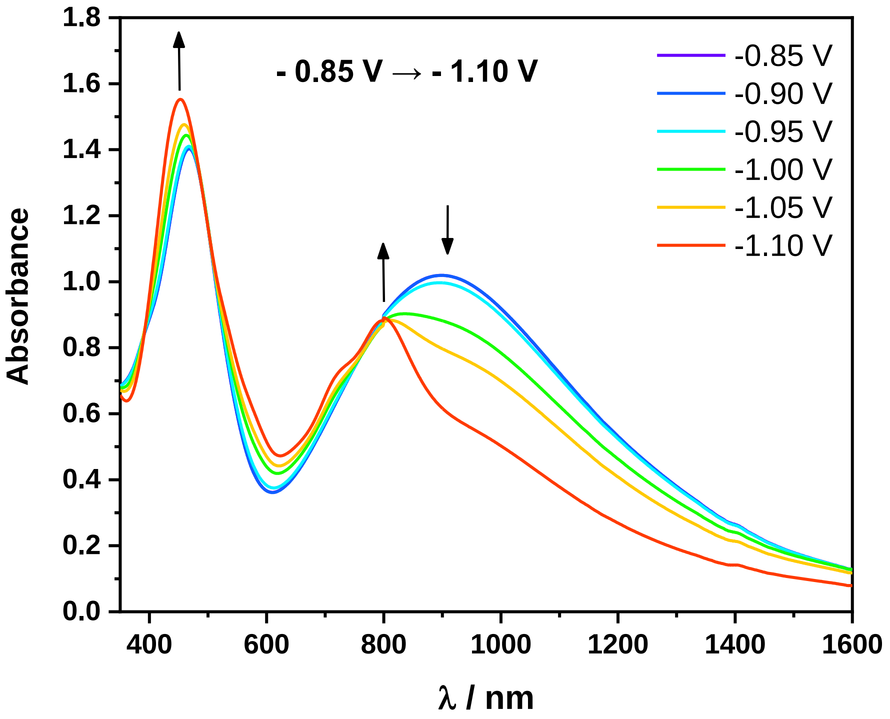

Figure S13. UV-vis–NIR spectra of a thin film of **poly(G-DTP-Bu-NDI)**, deposited on an ITO electrode and registered for decreasing electrode potentials in the range from -0.85 V to -1.10 V. Electrolyte 0.1 M Bu_4NBF_4/ACN, E vs. Fc/Fc^+.

Table S3. UV-vis–NIR spectroscopic data obtained for **poly(G-DTP-Bu-NDI)** and **poly(G-DTP-G-NDI)**, in its neutral, radical anion, dianion, radical cation, and dication states.

Polymer	λ_{max} [nm]				
	neutral	radical anion	dianion	radical cation	dication
**poly(G-DTP-Bu-NDI)**	467, 898	453, 898	—	568, 1025	651
**poly(G-DTP-G-NDI)**	473, 955	451, 801	446	565, 1036	650

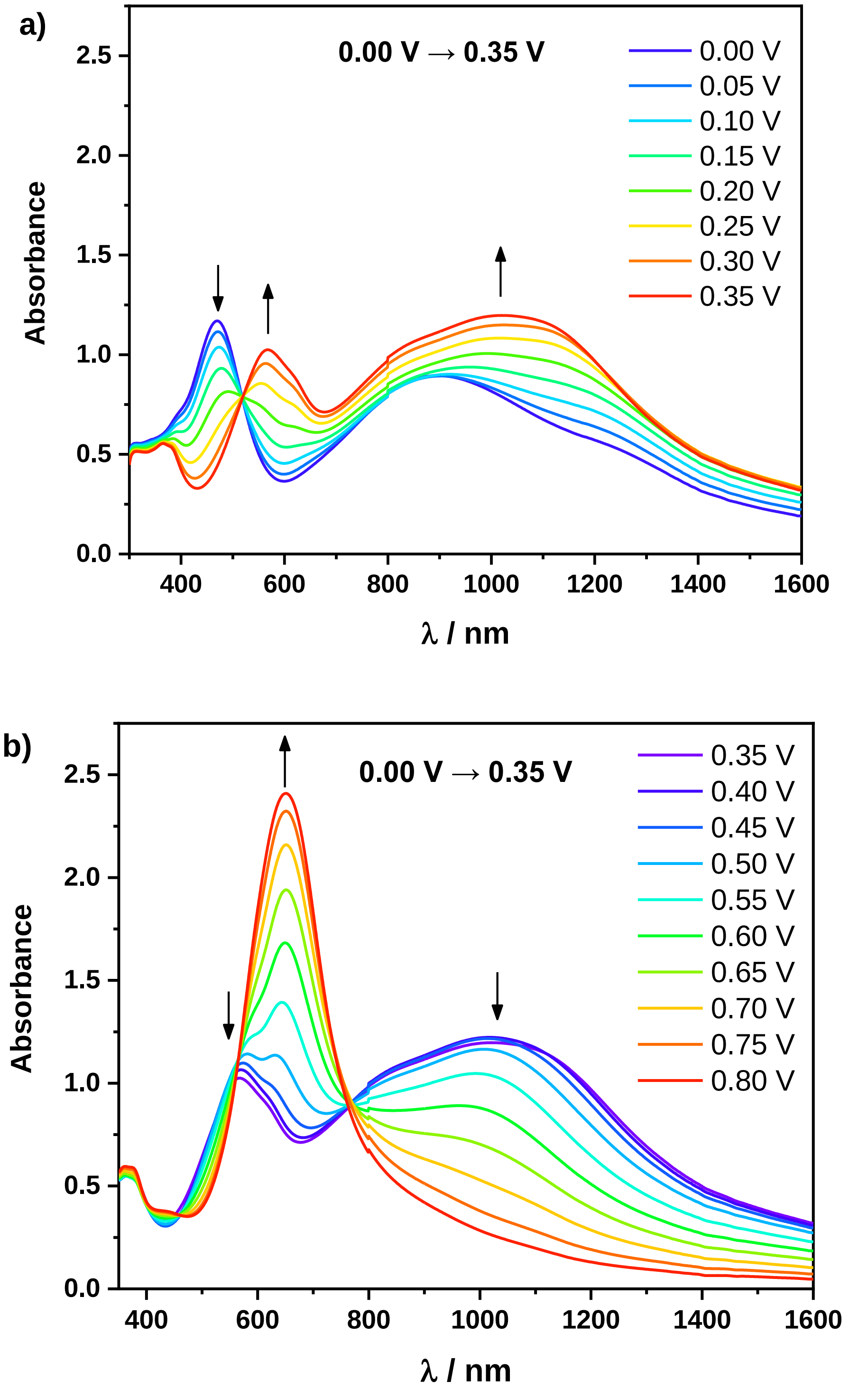

Figure S14. UV-vis–NIR spectra of a thin film of **poly(G-DTP-Bu-NDI)**, deposited on an ITO electrode and registered for increasing electrode potential: (a) potentials range from 0.0 V to +0.35 V; (b) from +0.35 to +0.80 V. Electrolyte 0.1 M Bu_4NBF_4/ACN, E vs. Fc/Fc^+.

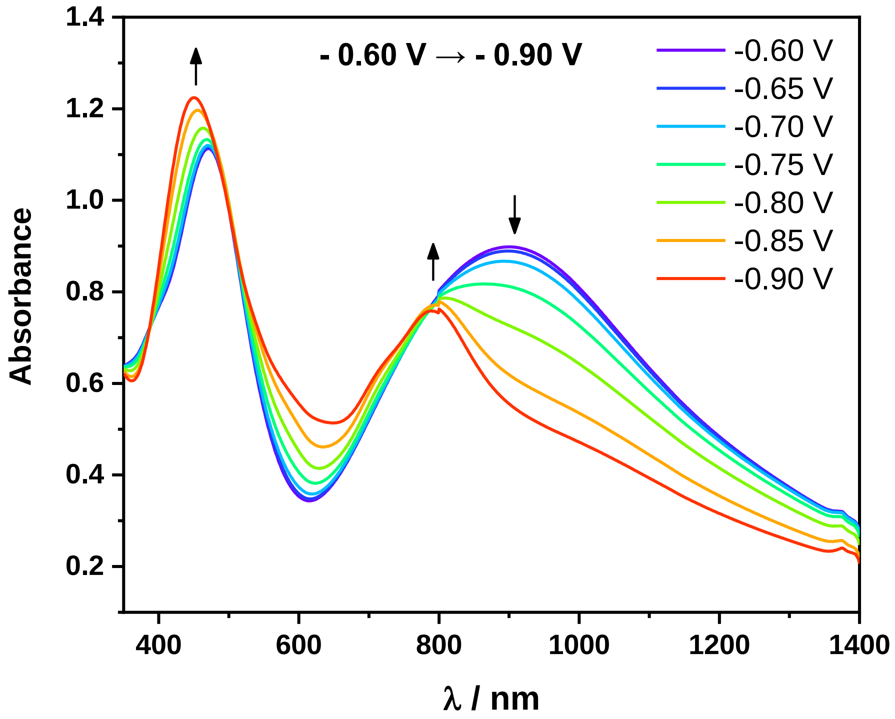

Figure S15. UV-vis-NIR spectra of thin films of **poly(G-DTP-Bu-NDI)** electrochemically deposited on an ITO substrate, recorded in 0.1 M NaCl aqueous electrolytes for decreasing electrode potentials from -0.60 V to -0.90 V vs. Ag/AgCl.

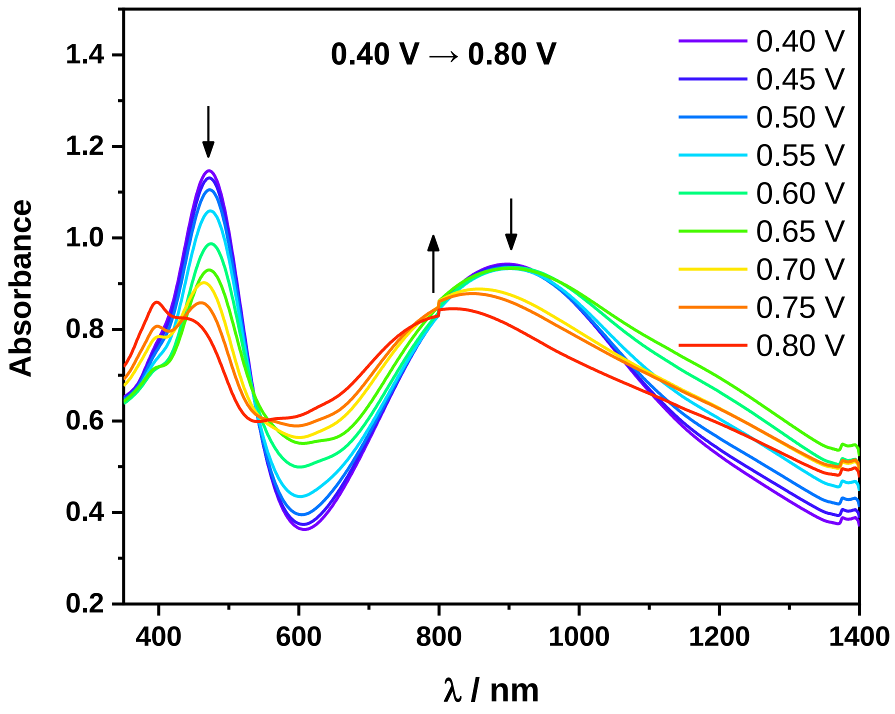

Figure S16. UV-vis-NIR spectra of thin films of **poly(G-DTP-Bu-NDI)** electrochemically deposited on an ITO substrate, recorded in 0.1 M NaCl aqueous electrolytes for increasing electrode potentials from +0.40 V to 0.80 V vs. Ag/AgCl.

4. OECTs fabrication characteristics

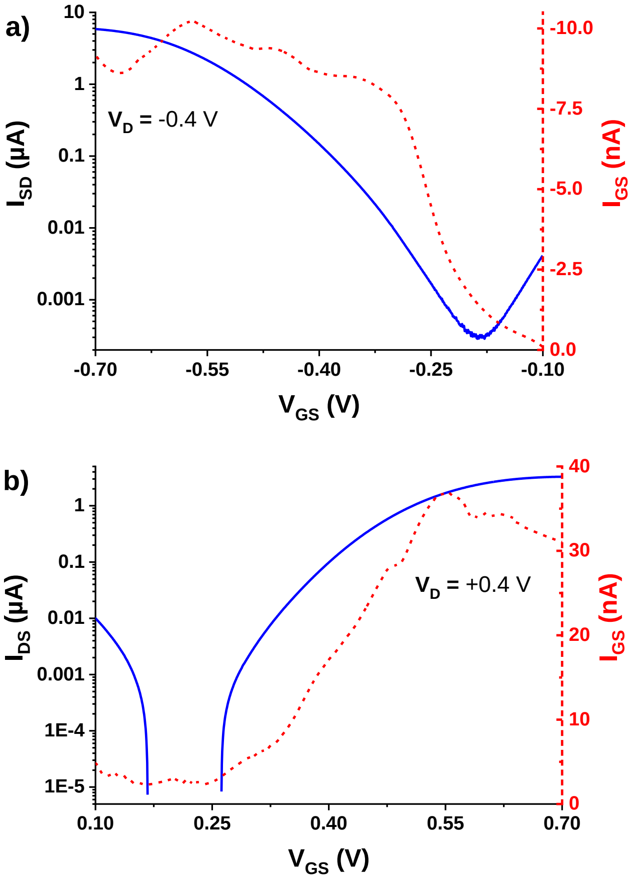

Figure S17. The logarithmic representation of selected transfer characteristics (Figures 7 c and 7d) along with corresponding gate currents for **poly(G-NDI-G-DTP)** transistors (obtained by electropolymerization with 2.25 µC charge) in p-type and n-type operation.

5. References

[1] T. Cardeynaels, M. K. Etherington, S. Paredis, A. S. Batsanov, J. Deckers, K. Stavrou, D. Vanderzande, A. P. Monkman, B. Champagne, W. Maes, *J. Mater. Chem. C* **2022**, *10*, 5840–5848.

[2] S. Förtsch, A. Vogt, P. Bäuerle, *Journal of Physical Organic Chemistry* **2017**, *30*, e3743.

[3] S. Förtsch, P. Bäuerle, *Polym. Chem.* **2017**, *8*, 3586–3595.

[4] D. Popović, I. Ata, J. Krantz, S. Lucas, M. Lindén, E. Mena-Osteritz, P. Bäuerle, *J. Mater. Chem. C* **2017**, *5*, 9920–9928.

[5] R. Rybakiewicz, E. D. Glowacki, L. Skorka, S. Pluczyk, P. Zassowski, D. H. Apaydin, M. Lapkowski, M. Zagorska, A. Pron, *Chem. Eur. J.* **2017**, *23*, 2839–2851.

[6] S. H. Jung, J.-W. Lee, H.-J. Kim, *Supramolecular Chemistry* **2016**, *28*, 634–639.
